# FAS-controlled T cells drive lymphoproliferation through glycolysis without effector differentiation

**DOI:** 10.70962/jhi.20250233

**Published:** 2026-05-13

**Authors:** Maria Elena Maccari, Christoph König, Geoffroy Andrieux, Patrick Kury, Sarah A. Berger, Jasmin Mann, Beth Kelly, Simon Völkl, Chenglong Huang, Martin Helmstädter, Simon Lagies, Marco Fischer, Francesc Baixauli, Oliver Gorka, Olaf Groß, Markus Hufnagel, Sarah Salou, Susan Farmand, Sabine Heine, Volker Schuster, Wolfgang Willenbacher, Gregor Dückers, Bodo Grimbacher, Klaus Warnatz, Friedrich G. Kapp, Myriam Lorenz, Miriam Groß, Jens Wittner, Roland Elling, Luca Scorrano, Iwona A. Koenig, Bertram Bengsch, Carsten Speckmann, Bernd Kammerer, Melanie Börries, Klaus Schwarz, Johannes Hertel, Erika L. Pearce, Stephan Ehl, Ramon I. Klein Geltink, Anne Rensing-Ehl

**Affiliations:** 1 Institute for Immunodeficiency, Center for Chronic Immunodeficiency, Medical Center-University of Freiburg, Faculty of Medicine, University of Freiburg, Freiburg, Germany; 2 Department of Pediatric Hematology, Oncology and Stem Cell Transplantation, Children’s Hospital, Medical Center – University of Freiburg, Faculty of Medicine, University of Freiburg, Freiburg, Germany; 3 Faculty of Biology, University of Freiburg, Freiburg, Germany; 4 Institute of Medical Bioinformatics and Systems Medicine, Medical Center - University of Freiburg, Faculty of Medicine, University of Freiburg, Freiburg, Germany; 5 Bloomberg-Kimmel Institute of Immunotherapy, Department of Oncology, Johns Hopkins University School of Medicine, Baltimore, MD, USA; 6 Department of Internal Medicine 5, Hematology and Oncology, Friedrich-Alexander-Universität Erlangen-Nürnberg and Universitätsklinikum Erlangen, Erlangen, Germany; 7 Department for Psychiatry and Psychotherapy, University Medicine Greifswald, Greifswald, Germany; 8 Department of Medicine IV, University Freiburg Medical Center, Faculty of Medicine, University of Freiburg, Freiburg, Germany; 9 EMcore, Renal Division, Department of Medicine, University Freiburg Medical Center, Faculty of Medicine, University of Freiburg, Freiburg, Germany; 10 Core Competence Metabolomics, Hilde-Mangold-Haus, University of Freiburg, Freiburg, Germany; 11 Department of Allergology and Clinical Immunology, Children’s Hospital of Eastern Switzerland, St. Gallen, Switzerland; 12 Department of Biomedicine, University of Basel, Basel, Switzerland; 13 Institute of Neuropathology, Faculty of Medicine, Medical Center, University of Freiburg, Freiburg, Germany; 14 Centre for Integrative Biological Signalling Studies and Signalling Research Centre, Albert-Ludwigs University, Freiburg, Germany; 15 Division of Pediatric Rheumatology and Clinical Infectious Diseases, Department of General Pediatrics, Adolescent Medicine and Neonatology, Center for Pediatrics, Medical Center and Medical Faculty, University of Freiburg, Freiburg, Germany; 16 Division of Pediatric Stem Cell Transplantation and Immunology, Department of Pediatric Hematology and Oncology, University Medical Center Hamburg-Eppendorf, Hamburg, Germany; 17 Pediatric Oncology and Hematology, Children’s Hospital Medical Center, University Clinics Homburg, Homburg, Germany; 18 Medical Faculty and Hospital for Children and Adolescents, University of Leipzig, Leipzig, Germany; 19 Internal Medicine V: Hematology & Oncology, Innsbruck Medical University, Innsbruck, Austria; 20 Syndena GmbH, Connect to Cure, Innsbruck, Austria; 21 Helios Children’s Hospital, Krefeld, Germany; 22 Clinic of Rheumatology and Clinical Immunology, Center for Chronic Immunodeficiency, Medical Center, Faculty of Medicine, Albert-Ludwigs-University of Freiburg, Freiburg, Germany; 23 German Center for Infection Research, Satellite Center Freiburg, Freiburg, Germany; 24 RESIST – Cluster of Excellence 2155 to Hanover Medical School, Satellite Center Freiburg, Freiburg, Germany; 25 Institute for Transfusion Medicine, University of Ulm, Ulm, Germany; 26 Department of Pediatrics and Adolescent Medicine, Faculty of Medicine, University Medical Center, University of Freiburg, Freiburg, Germany; 27 Veneto Institute of Molecular Medicine and Department of Biology, University of Padua, Padua, Italy; 28 Department of Microbiology and Immunology, SUNY Upstate Medical University, Syracuse, NY, USA; 29 Clinic for Internal Medicine II, University Medical Center Freiburg, Faculty of Medicine, Freiburg, Germany; 30 German Cancer Consortium, Partner Site Freiburg, A Partnership Between Deutsche Krebsforschungszentrum and Medical Center, University of Freiburg, Freiburg, Germany; 31 Institute for Clinical Transfusion Medicine and Immunogenetics Ulm, German Red Cross Blood Service Baden-Wuerttemberg-Hessen, Ulm, Germany; 32 German Center for Cardiovascular Diseases, Partner Site Greifswald, Greifswald, Germany; 33 BC Children’s Hospital Research Institute, Vancouver, Canada; 34 Department of Pathology and Laboratory Medicine, University of British Columbia, Vancouver, Canada; 35 Edwin S.H. Leong Centre for Healthy Aging, University of British Columbia, Vancouver, Canada; 36 Department of Basic and Translational Research, BC Cancer Research Centre, Vancouver, Canada

## Abstract

Lymphoproliferation in autoimmune lymphoproliferative syndrome (ALPS) due to FAS deficiency is driven by highly proliferative FAS-controlled T cells (FCT) with a distinct molecular signature. Activating signals and metabolic fuels of their proliferation are poorly understood. Lymphoproliferation caused by proliferative T cells is also a hallmark of acute EBV infection. In these antiviral T cells, a metabolic switch to glycolysis underpins effector differentiation and IFNγ translation. Here, we used EBV-induced CD8 effector T cells as a benchmark to characterize FCT metabolism. Metabolic assays, RNA sequencing, and in silico computational analysis revealed that FCT are as highly glycolytic as EBV-induced effector T cells, but this metabolic program is uncoupled from T-BET expression and IFNγ production. In contrast to virus-activated T cells, FCT showed mitochondrial hyperpolarization and elevated reactive oxygen species production. These findings support a model of FCT lymphoproliferation, in which activating signals strongly enhance glycolysis but do not induce classical effector differentiation.

## Teaser

In FAS deficiency, lymphoproliferation is driven by FAS-controlled T cells (FCT), yet underlying metabolic programs remain unclear. Using EBV-activated effector T cells as a benchmark, Maccari et al. show that FCT display comparably heightened glycolysis, but a metabolic program uncoupled from T-BET expression and IFNγ production.

## Graphical Abstract

**Figure:**
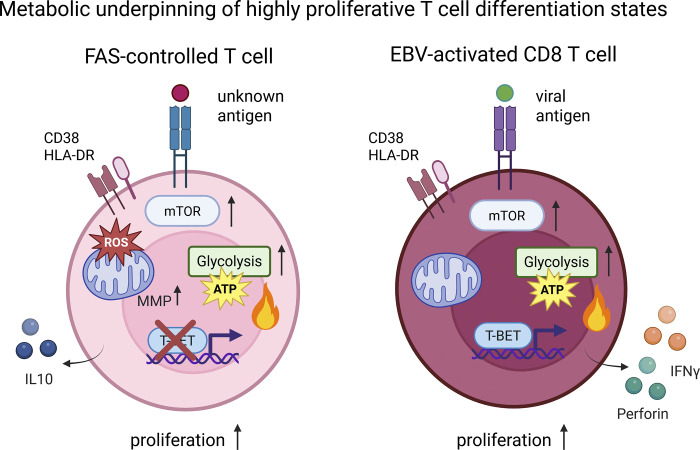

## Introduction

FAS-controlled T cells (FCT) represent a recently defined, physiological T cell subset stringently controlled by FAS-mediated apoptosis (1). FCT are highly proliferative and characterized by a distinct phenotype and transcriptome differing from other known T cell differentiation subsets (1). They can be found among mature CD4, CD8, and T cell receptor (TCR)αβ^+^CD4^−^CD8^−^ double-negative T cells (DNT) and are defined via flow cytometry by a CD38^high^CD45RA^high^CD45R B220^+^ phenotype (1). In healthy individuals, FCT make up <1% of TCRαβ^+^ T cells, whereas they massively accumulate, mostly as DNT, in patients with autoimmune lymphoproliferative syndrome (ALPS), caused by genetic defects in the FAS signaling pathway (2, 3, 4, 5). Thus, the majority of ALPS-DNT are FC-DNT (1). It was previously shown that human FC-DNT share TCR CDR3β sequences with phenotypically identical FC CD4 and FC CD8, suggesting that these FC CD4/8 can transition to FC-DNT by loss of co-receptor expression (1, 6). TCR CDR3β sequence comparisons suggested a predominant CD8 origin of DNT in ALPS (7).

Lymphoproliferative manifestations in ALPS patients are caused by uncontrolled proliferation of activated FCT cells, which cannot be eliminated via FAS-induced apoptosis. The highly activated state is reflected by their high CD38, HLA-DR, and Ki-67 expression (1). Strong in vivo proliferation leading to lymphadenopathy and splenomegaly is also a feature of CD8 T cells responding to acute Epstein–Barr virus (EBV) infection. In ALPS patients, the driving signals of FCT proliferation are currently poorly understood. Specific infectious triggers of lymphoproliferative manifestations in ALPS patients have not been reported (8, 9, 10). Notably, Fas-deficient mice develop lymphadenopathy with accumulation of B220^+^ DNT under germ-free conditions (11). Increased CD3ζ phosphorylation was demonstrated in B220^+^ DNT of Fas-deficient mice, indicating active TCR signaling (12, 13). In addition, elevated FCT frequencies in patients with CTLA-4 deficiency (1) or STAT3-activating mutations support a role for CD28 and STAT3-activating cytokines in FCT activation/expansion. Finally, FCT show active mTOR signaling (1, 14), which can be triggered by TCR, co-stimulatory molecules, and/or cytokines. Consistently, FCT are highly susceptible to mTOR inhibition and rapidly decline under rapamycin treatment, which represents a targeted therapy for ALPS patients (15). In summary, FCT are highly active, proliferating cells—both in healthy individuals and in FAS-deficient patients—and exhibit signs of TCR activation. These characteristics closely resemble those of virus-activated CD8 T cells.

TCR activation and co-stimulation initiate sets of signaling cascades that promote T cell exit from quiescence (16). The mTOR pathway is crucial for early metabolic changes associated with T cell activation and differentiation (17, 18, 19). A crucial metabolic change is an increase in glucose metabolism. CD28-mediated signaling controls the expression of the plasma membrane glucose transporter, Glut1 (20). In addition to increasing glucose import, activated T cells engage aerobic glycolysis to generate ATP and metabolic intermediates required for cell growth. This glycolytic switch has been shown mainly in murine effector T cells under controlled infectious conditions. Enhanced glycolysis not only fuels proliferation but directly promotes IFNγ translation and thus effector function (21). Indeed, T cells activated without glucose have defective effector function (22). If T cells are continuously stimulated, as in chronic viral infection, they can become exhausted, characterized by attenuated glycolytic metabolism, mitochondrial dysfunction, and impaired functionality (23, 24). Effector T cells also increase their mitochondrial mass but show fissed rather than the fused mitochondria characteristic for memory T cells, which rely on oxidative phosphorylation (OXPHOS) as their major source of energy (25). Effector T cells generate reactive oxygen species (ROS) via the mitochondrial respiratory chain or dedicated ROS-producing enzymes such as NADPH oxidases (26). These ROS have important signaling function but in excess can cause oxidative stress and cellular damage (25, 27, 28).

To understand the metabolic processes underlying abnormal lymphoproliferation in ALPS, we studied the metabolic profile of the highly active FCT cells using metabolic assays, RNA-sequencing analysis, as well as theoretical modeling via genome-scale metabolic reconstruction and constraint-based modeling (29, 30). As a benchmark for strongly in vivo–activated effector T cells, we compared the FCT profile to that of T cells activated during primary EBV infection. Resting T cells from healthy individuals served as controls. The identified similarities and differences provide insights into the metabolic processes fueling lymphoproliferation and may in the future open novel treatment options targeting the metabolic basis of lymphoproliferative diseases.

## Results

### ALPS-DNT are as highly activated and proliferative as acutely EBV-stimulated CD8 T cells

**Figure 1.**
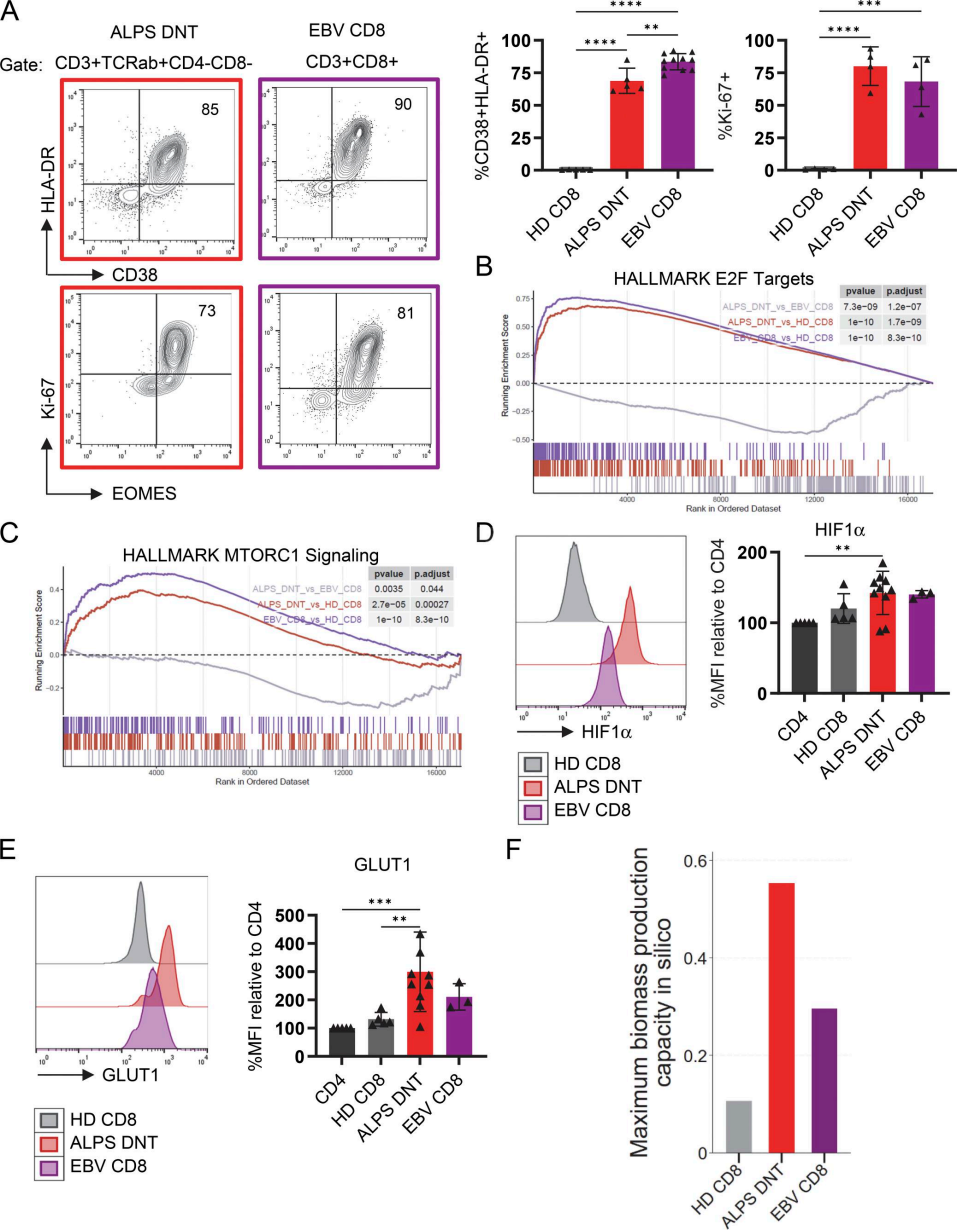
**DNT in ALPS are as highly activated and proliferative as acutely EBV-stimulated CD8 T cells. (A)** Representative flow cytometry plots showing CD38, HLA-DR, Ki-67, and EOMES expression in DNT of an ALPS patient and in CD8 T cells of an individual with acute EBV infection. Summary graph showing percentages of HLA-DR^+^CD38^+^ cells among total CD8 T cells of 11 individuals with acute EBV, DNT of 5 untreated ALPS patients, and CD8 T cells of 5 HD. **(B and C)** Enrichment score plots for the Hallmark gene sets “E2F Targets” (B) (normalized enrichment score [NES]: ALPS-DNT vs. EBV-CD8 −2.01; ALPS-DNT vs. HD-CD8 2.98; EBV-CD8 vs. HD-CD8 3.38) and “mTORC1 Signaling” (C) (NES: ALPS-DNT vs. EBV-CD8 −1.44; ALPS-DNT vs. HD-CD8 1.71; EBV-CD8 vs. HD-CD8 2.21) generated with RNA-sequencing data of sorted ALPS-DNT (CD3^+^TCRαβ^+^CD4^−^CD8^−^CD45RA^+^CD38^+^) from three untreated ALPS patients, activated CD8 T cells (CD8^+^CD38^+^) from three individuals with acute EBV infection, and naïve and early-differentiated CD8 T cells (CD8^+^CD28^+^CD57^−^) from three HD. **(D and E)** Representative histogram and summary plot of flow cytometric quantification of HIF1α (D) and GLUT1 expression (E). The median fluorescence intensities (MFIs) were normalized to those of the respective CD4 subset to allow for comparisons across multiple acquisition dates. **(F)** Maximum biomass flux values (mmol/gDW/h) predicted by FBA across T cell models based on constraint settings derived from RNA-sequencing data of sorted ALPS-DNT, EBV-CD8, and HD-CD8 (three samples/population). In all figures, statistical comparisons were conducted using the one-way ANOVA test for multiple comparisons. Only statistically significant differences between subsets are shown (**P < 0.01, ***P < 0.001, and ****P < 0.0001). Bars and error bars indicate mean ± SD.

**Figure S1.**
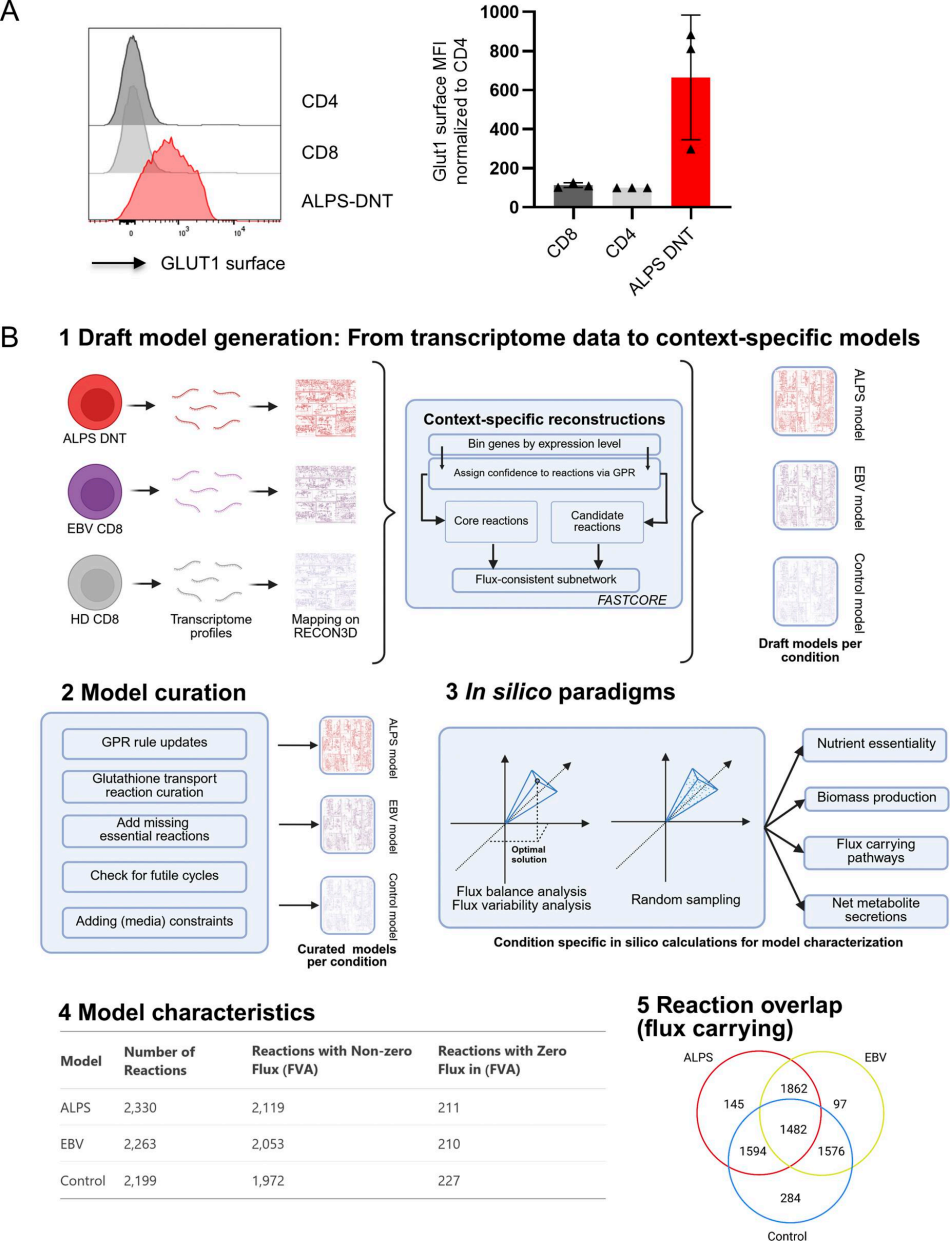
**GLUT1 surface expression and workflow for the generation and in silico analysis of context-specific metabolic models for ALPS-DNT, EBV-CD8, and HD-CD8 T cell populations. (A)** Representative flow cytometry histogram showing surface GLUT1 expression on CD4, CD8, and ALPS-DNT of an untreated ALPS patient. Summary plot showing data of three independent untreated ALPS patients. **(B)** Transcriptomic profiles from three T cell subsets—ALPS-DNT, EBV-CD8, and HD-CD8—were integrated with the generic human metabolic network (Recon3D). The fastCore algorithm was used to generate draft, context-specific models based on gene expression levels and associated gene-protein-reaction (GPR) rules. These draft models were then manually curated by refining GPRs, updating key transport reactions such as for glutathione, adding essential metabolic functions, and checking for possible futile cycles. Finally, the curated models were constrained with experimental data and analyzed using flux balance analysis (FBA), flux variability analysis (FVA), and random sampling to characterize condition-specific metabolic phenotypes, including nutrient essentiality, biomass production, and metabolite secretion profiles.

To study the metabolic basis of T cell–driven lymphoproliferation in ALPS, we focused on TCRαβ^+^CD4^−^CD8^−^ DNT (ALPS-DNT), which in many untreated ALPS patients consist of >90% FC-DNT. As a benchmark for highly in vivo–activated T cells, we compared them to CD8 T cells of individuals with lymphoproliferation driven by acute primary EBV infection (EBV-CD8) in the absence of a known genetic predisposition. As expected for bona fide CD8 effector cells, human CD8 responding to EBV were highly activated, proliferative, and expressed CD38, HLA-DR, EOMES, and Ki67 (Fig. 1 A and Table S1) (31). Bulk RNA-sequencing analysis of sorted T cell populations revealed highly expressed cell cycle–related genes targeted by the transcription factor (TF) E2F in ALPS-DNTs compared to CD8 T cells of healthy individuals (healthy donor [HD]-CD8) (Fig. 1 B). Interestingly, expression levels of activation and proliferation markers as well as cell division–associated genes were similarly elevated albeit not identical in ALPS-DNT compared to EBV-CD8, (Fig. 1, A and B). Transcripts associated with mTOR signaling were also increased in both cell populations (Fig. 1 C). Consistently, flow cytometry revealed increased expression of the mTOR downstream targets HIF1α and GLUT1 in ALPS-DNT and less pronounced in EBV-CD8 (Fig. 1, D and E) when compared to HD-CD8. GLUT1 expression was not only increased intracellularly but also on the cell surface (Fig. S1 A). To evaluate the capacity of ALPS-DNT, EBV-CD8, and HD-CD8 for biomass production in silico, we performed flux balance analysis (FBA) with RNA-sequencing data as input using the generic human biomass reaction from Recon3D (29), which models the stoichiometric demands of major biomass precursors, including amino acids, nucleotides, lipids, and cofactors (Fig. S1 B and Materials and methods section). ALPS-DNT and EBV-CD8 models showed greatly increased biomass production capacity in silico compared to HD-CD8 (Fig. 1 F) after specifying metabolic constraints according to the cell-specific transcriptomic profiles (30). In conclusion, ALPS-DNT showed a similar degree of activation, mTOR signaling, biomass production capacity, and proliferation compared to acutely EBV-stimulated CD8 T cells.

### ALPS-DNT are as highly glycolytic as acutely EBV-stimulated CD8 T cells

**Figure 2.**
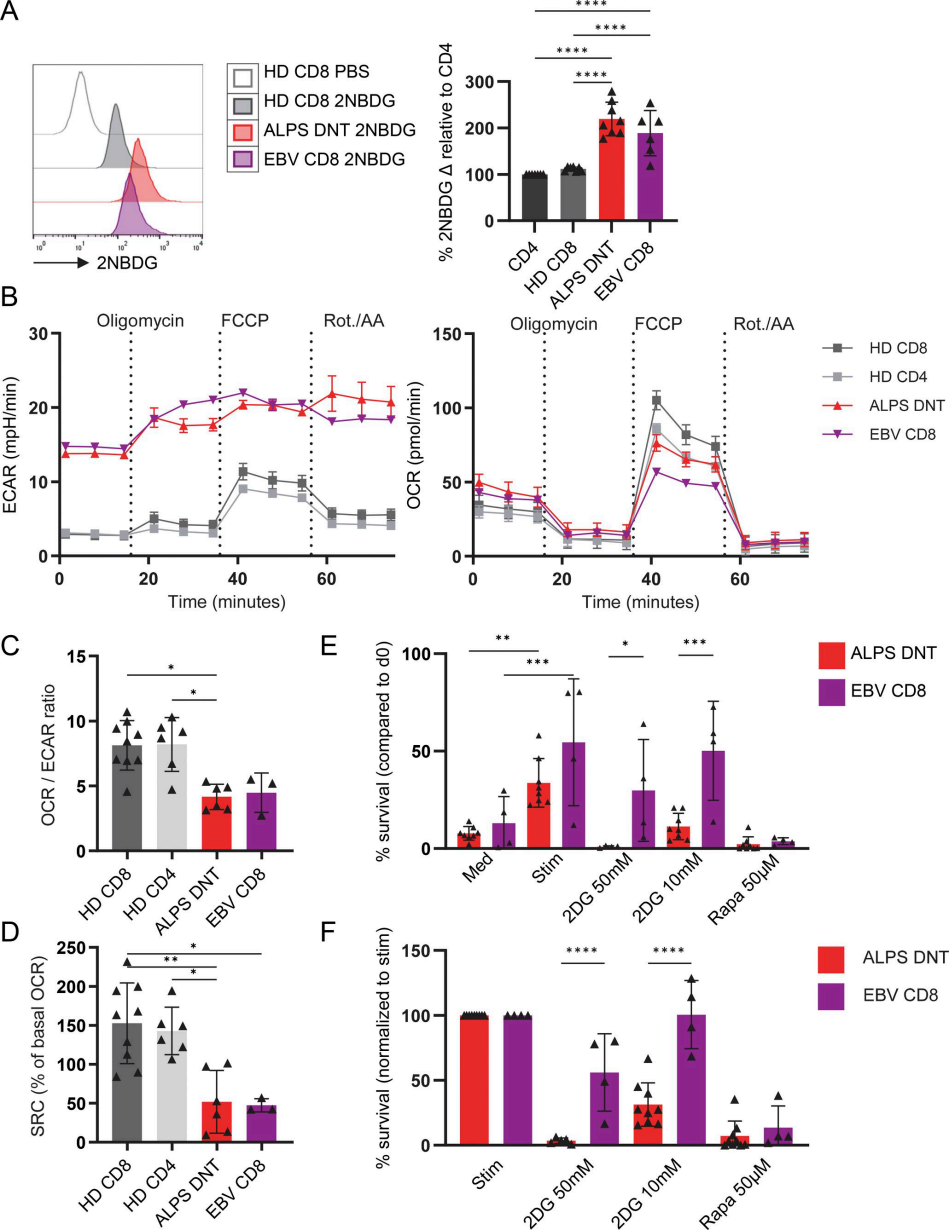
**DNT in ALPS are as highly glycolytic as acutely EBV-stimulated CD8 T cells but are more sensitive to glucose deprivation. (A)** PBMC of ALPS patients (*n* = 8), individuals with acute EBV infection (*n* = 6), and noninfected HD (*n* = 7) were incubated with fluorescent 2-NBDG, and its uptake was quantified by flow cytometry and subsequent gating on the populations of interest (ALPS-DNT: CD3^+^TCRαβ^+^CD4^−^CD8^−^), EBV-CD8 (CD3^+^CD8^+^), HD-CD8, and HD-CD4 (CD3^+^CD8^+^ and CD3^+^CD4^+^). To allow for comparisons across multiple acquisition dates, the delta MFI between PBS-incubated versus 2-NBDG–incubated CD4 T cells was set as 100%, and the delta MFIs of the target populations were calculated relative to this value. **(B)** Representative Seahorse curves for ECAR and OCR for the indicated sorted cell populations (six ALPS-DNT, three EBV-CD8, nine HD-CD8, and six HD-CD4) before and after the addition of carbonyl cyanide-p-trifluoromethoxy phenylhydrazone (FCCP), rotenone (Rot.), and antimycin A (AA). **(C)** Summary plot of OCR/ECAR ratios of all Seahorse experiments. **(D)** Summary plot of spare respiratory capacity (SRC), calculated as the difference between the cells’ maximal respiration (OCR peak after adding FCCP) and its basal respiration (baseline OCR), of all Seahorse experiments. **(E)** Survival of ALPS FC-DNT or of EBV-CD8 (gated as follows: ALPS-DNT: CD3^+^TCRαβ^+^CD4^−^CD8^−^CD45RA^+^CD38^+^, EBV-CD8: CD3^+^CD8^+^CD38^+^) among PBMC incubated with medium (Med), allogenic monocyte-derived DC at a ratio 1:1 plus IL-21 100 ng/ml and IL-10 100 ng/ml (Stim), or with the Stim condition plus 2DG or rapamycin (Rapa) at the indicated concentrations. Absolute cell counts of annexin V cells per well after 18-h incubation were determined by flow cytometry. Survival was calculated as (cell count day 1/cell count day 0)*100. Summary plot showing survival normalized to day 0 under different conditions. **(F)** Summary plot showing survival normalized to the Stim condition. In all figures, statistical comparisons were conducted using the one-way ANOVA test for multiple comparisons. Only statistically significant differences between subsets are shown (*P < 0.05, **P < 0.01, ***P < 0.001, and ****P < 0.0001). Bars and error bars indicate mean ± SD. MFI, median fluorescence intensity.

**Figure S2.**
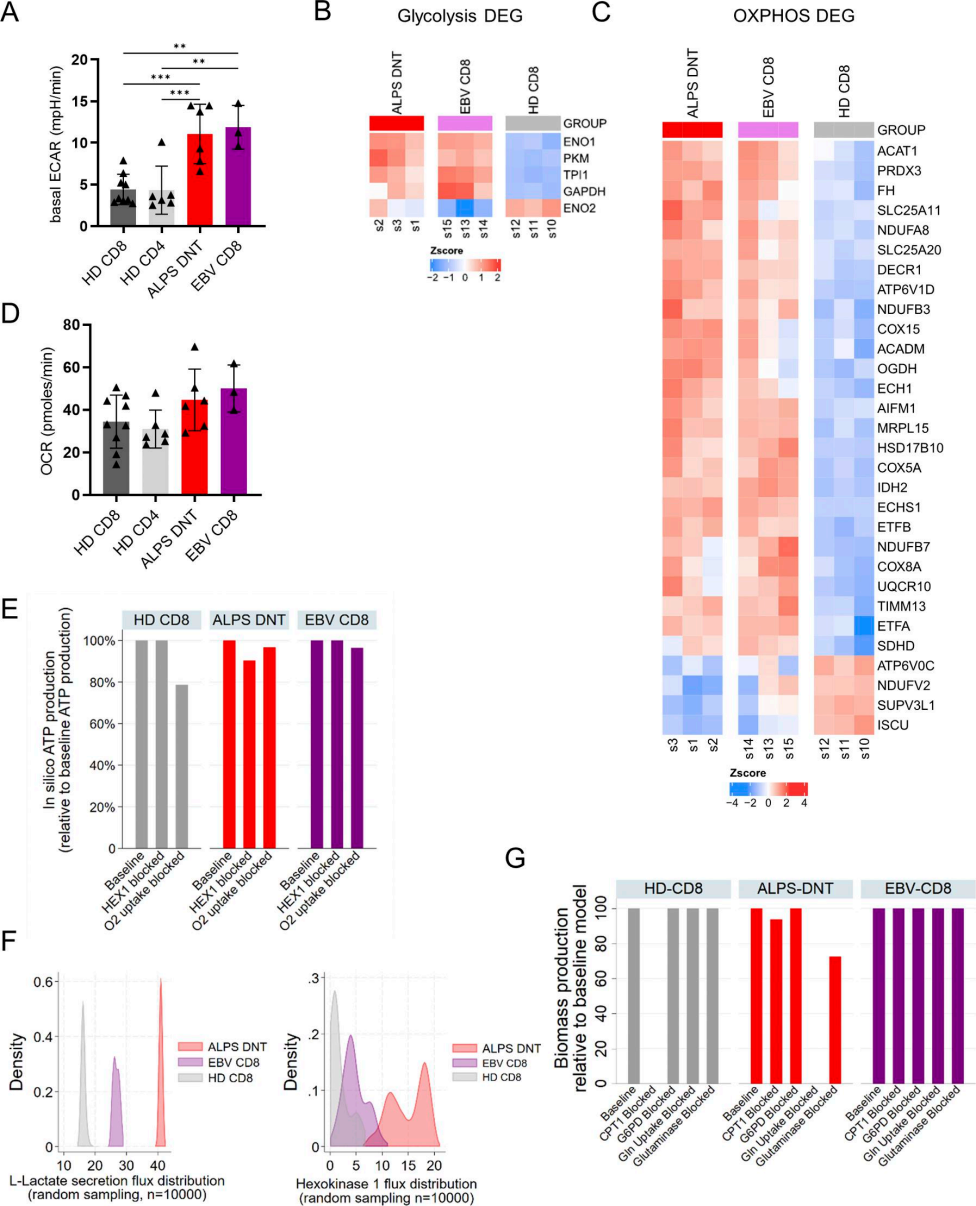
**Glycolysis and OXPHOS in ALPS-DNT and EBV-CD8. (A)** Summary plot of basal ECAR values of all Seahorse experiments. **(B and C)** Expression levels of selected glycolysis-related enzymes (B) and OXPHOS-related genes (C) based on RNA-sequencing data from sorted ALPS-DNT (CD3^+^TCRαβ^+^CD4^−^CD8^−^), EBV-CD8 (CD3^+^CD8^+^CD38^+^), and HD-CD8 (CD3^+^CD8^+^) from three ALPS patients, three individuals with acute EBV infection, and three HD. The relative expression (Z-score) of genes is shown and color coded according to the legend. Rows are scaled to have a mean of 0 and SD of 1. **(D)** Summary plot of basal OCR values of all Seahorse experiments. **(E)** In silico ATP production capacity expressed relatively to the ATP production capacity to baseline models. Hexokinase reaction blocking in silico affected only ALPS-DNT model ATP production capacity, while simulating anoxic conditions (O^2^ uptake blocking) led only to a substantial loss in ATP production capacity in HD-CD8 models. This demonstrates the reliance of ALPS-DNT models on glycolysis and hexokinase activity in particular. **(F)** Kernel density plots for the glutathione synthetase flux distributions from uniform random flux sampling (*n* = 10,000), showing the higher L-lactate secretion and hexokinase fluxes (expressed in mmol/gDW/h) in ALPS-DNT models in comparison to EBV-CD8 and HD-CD8 models. **(G)** Biomass production in silico under various inhibitor simulations relative to the baseline model without any inhibitors applied. Inhibitor simulations were performed by blocking the biochemical reactions associated with the inhibited enzyme. Missing bars represent failed biomass (e.g., zero biomass flux) production. In A and D, statistical comparisons were conducted using the one-way ANOVA test for multiple comparisons. Only statistically significant differences between subsets are shown (**P < 0.01, ***P < 0.001). Bars and error bars indicate mean ± SD.

**Figure S3.**
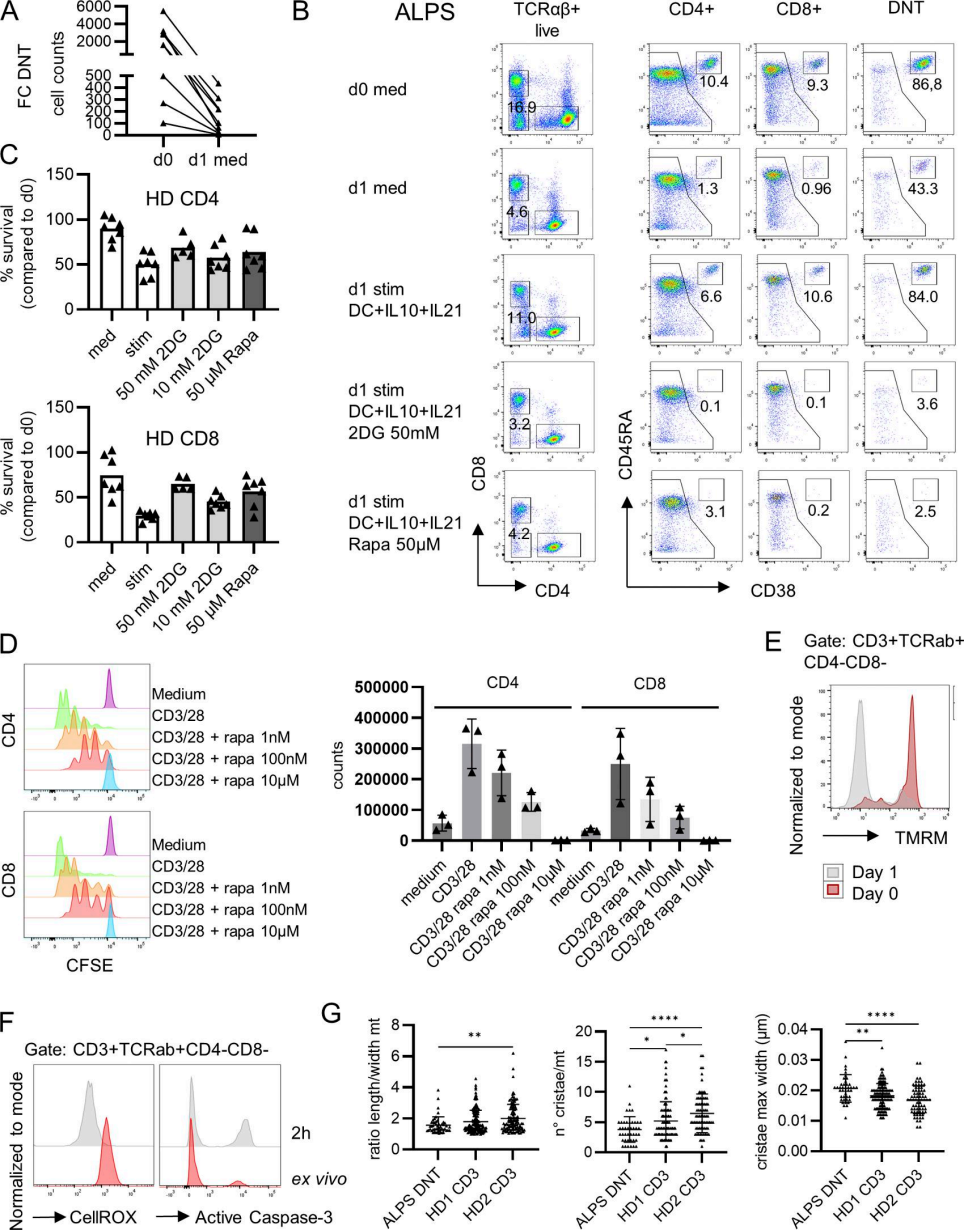
**FCT survival is highly dependent on glucose. (A)** The proportions and total cell counts of FC-DNT (CD38^+^CD45RA^+^ of total TCRαβ^+^ DNT) were determined in isolated PBMC directly after blood drawal and after incubation of PBMC with medium alone for 24 h at 37°C (*n* = 9). **(B)** Representative flow cytometry plots showing FC CD4, FC CD8, and FC-DNT of an ALPS patient at day (d) 0 (immediately after blood withdrawal and PBMC isolation) and after 18 h of in vitro incubation with medium (Med), allogenic monocyte-derived DC at a ratio 1:1 plus IL-21 100 ng/ml and IL-10 100 ng/ml (Stim), or with the Stim condition plus 2DG or rapamycin (Rapa) at the indicated concentrations. The figures indicate the percentages of the populations of interest. **(C)** Summary plot showing survival normalized to day 0 under the above conditions for conventional (non-FC) CD4 and CD8 T cells of 7 HD. **(D)** Representative histograms and summary plots showing CFSE proliferation of CD4 and CD8 cells after in vitro activation with or without rapamycin at the indicated concentrations. Mean and SD of three independent healthy individuals. **(E)** Representative flow cytometry histogram showing the TMRM drop in ALPS-DNT of an ALPS patient 24 h after in vitro incubation with medium (day 1) compared to the day of cell isolation (day 0). **(F)** Representative flow cytometry histogram showing the progressive decrease in CellROX expression and increase in active caspase-3 expression in ALPS-DNT of an ALPS patient 2 h after incubation with medium. **(G)** Summary graph showing the ImageJ-based analysis of electron microscopy images of mitochondria of sorted ALPS-DNT of one ALPS patient and sorted total CD3^+^ T cells of two HD. Displayed are the ratio between length and width of the mitochondria, the maximal width of the cristae (in µm), and the number of cristae per mitochondrion. Statistical comparisons were conducted using the one-way ANOVA test for multiple comparisons. Only statistically significant differences between subsets are shown (*P < 0.05, **P < 0.01, and ****P < 0.0001). Bars and error bars indicate mean ± SD.

Consistent with increased expression of the glucose transporter GLUT1, enhanced glucose uptake was documented by increased 2-[N-(7-nitrobenz-2-oxa-1,3-diazol-4-yl) amino]-2-deoxy-D-glucose (2-NBDG) fluorescence of both ALPS-DNT and EBV-CD8 (Fig. 2 A). Functional Seahorse analysis of sorted cell populations showed increased extracellular acidification rate (ECAR) as a proxy for baseline glycolysis and increased glycolytic reserve in both ALPS-DNT and EBV-CD8 compared to HD-CD8 (Fig. 2 B and Fig. S2 A). Consistent with increased glucose metabolism, RNA-sequencing revealed comparably increased expression of glycolytic enzymes, including GAPDH, TPI1, α-enolase, and PKM (Fig. S2 B). In contrast, baseline oxygen consumption rate (OCR) was not significantly increased despite upregulation of OXPHOS-related genes (Fig. S2, C and D), leading to a relative shift to glycolytic metabolism as indicated by a lower OCR/ECAR ratio in ALPS-DNT and EBV-CD8 compared to resting HD-CD8 (Fig. 2 C). Notably, both cell populations showed reduced spare respiratory capacity (Fig. 2 D), as previously described for acute effector T cells (32, 33). Mirroring the seahorse analysis, the in silico investigations of the three T cell populations revealed stronger dependence of ATP production on glycolysis in EBV-CD8 and ALPS-DNT versus HD-CD8, with in silico ATP production only marginally affected under simulated anoxic conditions for EBV-CD8 and ALPS-DNT (Fig. S2 E). Furthermore, in silico analysis indicated markedly increased L-lactic acid production capacity and hexokinase fluxes in ALPS-DNT in comparison to EBV-CD8, which in return showed higher glycolytic capacity than HD-CD8 (Fig. S2 F). Using in silico blockade of the hexokinase reaction led to a decrease in in silico ATP production in ALPS-DNT models but not in EBV-CD8 and HD-CD8 models, indicating higher metabolic flexibility in EBV-CD8 cells than in ALPS-DNT cells (Fig. S2 E). Furthermore, we performed in silico inhibitor modeling for fatty acid oxidation (FAO) (blocking CPT1), glutaminolysis (blocking glutamine uptake and the mitochondrial glutaminase, respectively), and the pentose phosphate pathway (blocking G6PD). Intriguingly, ALPS-DNT models were no longer able to produce biomass upon blocking glutamine uptake, while also showing reduced biomass production with mitochondrial glutaminase blocked (Fig. S2 G), highlighting a possibly high glutamine dependency. In contrast, blocking FAO had only minor effects on the ALPS-DNT in silico biomass production, while the HD-CD8 models could no longer support biomass production with blocked CPT1 (Fig. S2 G). However, the EBV-CD8 models were unaffected by any in silico inhibitor modeling. Taken together, our in silico inhibitor modeling supports the hypothesis that FCT rely on distinct metabolic pathways, including glutamine-dependent metabolism, whereas EBV-CD8 cells appear metabolically more flexible and less sensitive to perturbation of these pathways. Consequently, we hypothesized that this strong dependence on glycolytic metabolism could be targeted to reduce FCT survival. Since ALPS-DNT rapidly undergo apoptosis ex vivo (34 and Fig. S3 A), we first established culture conditions that improve ALPS-DNT survival and enable metabolic intervention. Co-culturing peripheral blood mononuclear cells (PBMC) with allogenic monocyte-derived dendritic cells (DC) in the presence of IL-10 and IL-21 (“Stim” in Fig. 2, E and F) improved 24-h survival of CD38^high^CD45RA^high^ ALPS-DNT and CD38^+^ EBV-CD8, as determined by flow cytometric quantification of viable cells. We then performed overnight survival assays in the presence of the glycolysis-blocking agent 2-deoxy-D-glucose (2DG) or the mTORC1 inhibitor rapamycin. While both subsets showed reduced survival upon rapamycin addition, cytokine/monocyte-derived DC-sustained ALPS-DNT exhibited lower survival than EBV-CD8 cells upon inhibition of glucose metabolism (Fig. 2, E and F; and Fig. S3 B). In contrast, total CD4 and CD8 cells of healthy individuals were not affected by rapamycin or glycolysis inhibition (Fig. S3 C). Of note, the effect of rapamycin on ALPS-DNT cells most likely involves cell survival, as these cells are non-proliferative in vitro. However, an additional effect on proliferation is likely, as illustrated for anti-CD3/28 in vitro–activated CD4 or CD8 T cells (Fig. S3 D). In conclusion, ALPS-DNT cells share a metabolic profile similar to that of EBV-stimulated CD8^+^ T cells, predominantly driven by glycolysis.

### ALPS-DNT show an increased mitochondrial membrane potential and elevated ROS production

**Figure 3.**
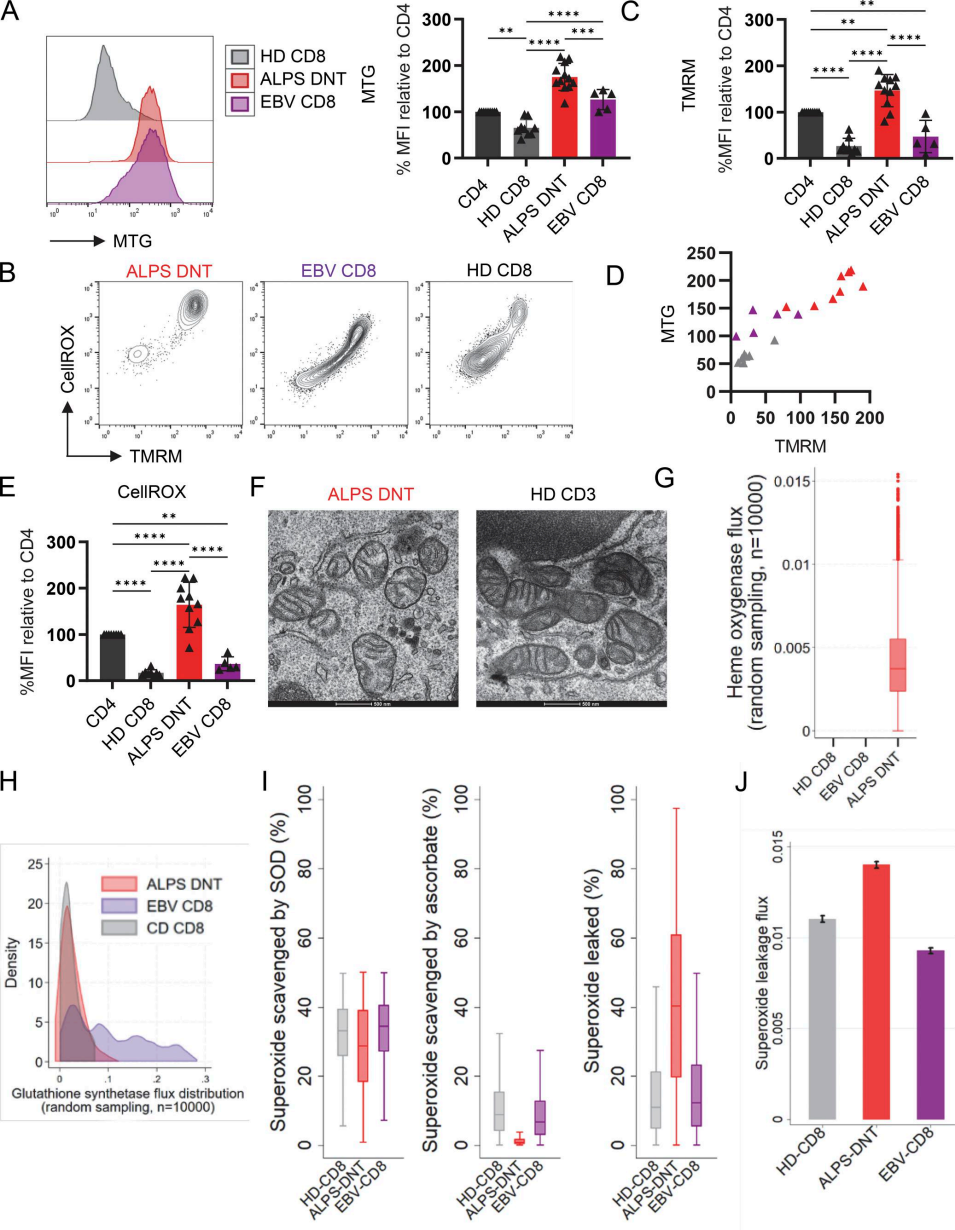
**ALPS-DNT show hyperpolarized mitochondria and high ROS production. (A)** Representative histogram and summary plot of flow cytometric measurement of MitoTracker Green (MTG) staining of PBMC gated for ALPS-DNT (*n* = 12), EBV-CD8 (*n* = 5), HD-CD8, and HD-CD4 (*n* = 9) as detailed in Fig. 2 A. Summary plots show the MTG median fluorescence intensities (MFIs) of the indicated populations normalized to CD4 as in Fig. 2 A. **(B)** Representative flow cytometry plots showing TMRM and CellROX fluorescence intensities in gated ALPS-DNT, EBV-CD8, and HD-CD8 after staining of total PBMC. **(C)** Summary plots for TMRM (ALPS *n* = 11; EBV *n* = 5; HD *n* = 9) MFIs in the indicated T cell subsets normalized to the MFI of CD4 as in Fig. 2 A. **(D)** Scatter plot showing MTG values (%MFI relative to CD4) and corresponding TMRM values (%MFI relative to CD4) for each sample (ALPS *n* = 8 in red; EBV *n* = 5 in purple; HD *n* = 7 in grey). **(E)** Summary plots for CellROX (ALPS *n* = 10; EBV *n* = 5; HD *n* = 8) MFI in the indicated T cell subsets normalized to the MFI of CD4 as in Fig. 2 A. **(F)** Representative electron microscopy images of mitochondria in sorted ALPS-DNT from one ALPS patient and sorted HD-CD8 from two HD. **(G)** Box plot for heme oxygenase flux from uniform random flux sampling (*n* = 10,000) in ALPS-DNT, EBV-CD8, and HD-CD8 models releasing carbon monoxide, biliverdin, and iron (FE2+). Only the ALSP-DNT model includes the heme oxygenase pathway and has therefore the capacity to release carbon monoxide. **(H)** Kernel density plots for the glutathione synthetase flux distributions from uniform random flux sampling (*n* = 10,000), showing the higher glutathione synthetase rates (expressed in mmol/gDW/h) in EBV-CD8 models in comparison to ALPS-DNT and HD-CD8 models. **(I and J)** Superoxide handling in silico across HD-CD8, ALPS-DNT, and EBV-CD8 genome scale models challenged by 2% superoxide production rate per consumed mol oxygen in the mitochondrial electron chain. Box plots indicate flux distributions from random flux sampling (*n* = 10,000), bars mean fluxes with 95% confidence intervals. SOD, sum of superoxide dismutase fluxes (e.g., mitochondrial, cytosolic, peroxisomal, and nuclear). In all figures, statistical comparisons were conducted using the one-way ANOVA test for multiple comparisons. Only statistically significant differences between subsets are shown (*P < 0.05, **P < 0.01, ***P < 0.001, and ****P < 0.0001). Bars and error bars indicate mean ± SD.

Mitochondria are critical contributors to cellular energy generation and macromolecule biosynthesis. Moreover, mitochondrial membrane potential (MMP) and ROS impact signaling, stemness, and viability/longevity (35). EBV-CD8 showed an increase in mitochondrial mass as determined by MitoTracker Green staining (Fig. 3 A). Mitochondrial mass of ALPS-DNT was even higher (Fig. 3 A). Interestingly, MMP and cellular ROS as determined by tetramethylrhodamine methyl ester (TMRM) and CellROX staining were highest and more homogenously elevated in ALPS-DNT compared to EBV-CD8 and HD-CD8 (Fig. 3, B–E). Importantly, these analyses were performed directly ex vivo within 1–2 h after blood collection and immediate PBMC isolation. MMP and ROS dropped over time ex vivo, concomitant with the induction of cell death (Fig. S3, D and E), suggesting that the high ROS levels ex vivo reflect the in vivo state and are not a consequence of impaired viability. As a possible cause of high MMP and ROS, we reasoned that altered mitochondrial ultrastructure and function could lead to impaired proton utilization (increased MMP) and electron leakage (increased ROS). Electron microscopy revealed that ALPS-DNT had fissed, round-shaped mitochondria as expected for effector T cells (Fig. 3 F) but with significantly increased width of the cristae and reduced numbers of cristae per mitochondrion (Fig. S3 F). Interestingly, when investigating condition-specific pathways in silico, the ALPS-DNT model identified heme oxygenase 1 (HMOX1)–mediated heme degradation leading to carbon monoxide production, a pathway linked to oxidative stress response (Fig. 3 G), which was not seen in HD-CD8 and EBV-CD8 models. This aligns with the experimental finding of elevated ROS in ALPS-DNT. Exploring further differences in ROS control and ROS response, the ALPS in silico models had lower glutathione synthetase fluxes in comparison to EBV-CD8 models as indicated by random flux sampling (Fig. 3 H), indicating potential limitations in effective ROS scavenging. To further explore the potential of ROS scavenging in silico, we induced 2% mitochondrial superoxide production per consumed mmol oxygen (see Materials and methods) and investigated superoxide detoxification pathways by random sampling (Fig. 3 I). In line with the experimental observations, ALPS-DNT models had the highest rate of superoxide leakage caused by a decreased superoxide detoxification by ascorbic acid (Fig. 3, I and J). As the models recover oxidized ascorbic acid via NADPH-dependent glutaredoxin activity, we tested then the maximum production potential of NADPH for each cell-specific model, finding that ALPS-DNT models had far lower NADPH production capacity (7.71 mmol/gDW/h) than EBV-CD8 (14.499 mmol/gDW/h) and HD-CD8 models (31.538 mmol/gDW/h). Collectively, FCT displayed an unusual increase in MMP and ROS accumulation, while the in silico models hint at limitations in core ROS scavenging pathways.

### FCT from HD show the same metabolic profile as ALPS-DNT

**Figure 4.**
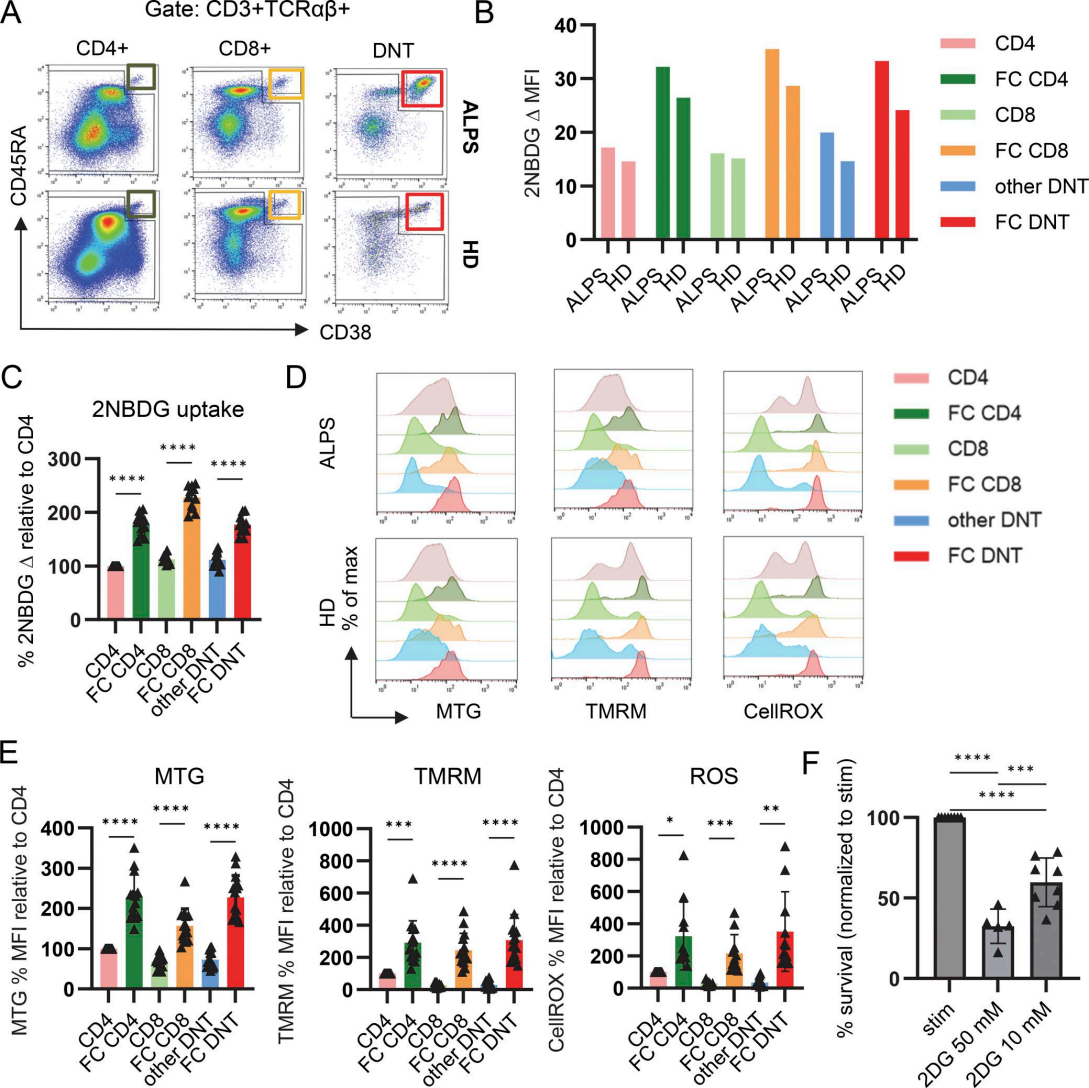
**FCT from** **HD** **show the same metabolic profile as ALPS-DNT. (A)** Representative flow cytometry plots showing the gating strategy for FAS-controlled (FC) CD38^+^CD45RA^+^ T cells among CD4, CD8, and DNT of an ALPS patient and a healthy individual (HD). **(B)** Representative experiment showing 2-NBDG uptake of FC and other T cells among CD4, CD8, and DNT in one ALPS patient and one HD. **(C)** Summary plot of 2-NBDG update in FCT and other T cells in healthy individuals (*n* = 12). The delta 2-NBDG median fluorescence intensity (MFI) was calculated by subtracting the MFI of PBS incubated cells from the MFI of 2-NBDG incubated cells. Delta MFI values were normalized to the delta MFI of CD4 T cells to allow for comparisons across multiple acquisition dates. **(D)** Representative flow cytometry histograms showing MTG, TMRM, and CellROX in the indicated T cell subsets of an ALPS patient and a HD. **(E)** Summary plots of MTG (*n* = 14), TMRM (*n* = 16), and CellROX (*n* = 11) median fluorescence intensities (MFIs) in FCT and conventional T cells of HD. The MFI values were normalized to that of CD4. **(F)** To investigate the effect of 2DG on in vitro survival of HD FC-DNT, PBMC of eight HD were incubated with medium (Med), with allogenic monocyte-derived DC at a ratio 1:1 plus IL-21 100 ng/ml and IL-10 100 ng/ml (Stim), or with the Stim condition plus 2DG 10 mM (*n* = 8) or 50 mM (*n* = 5) for 18 h. Cell counts of viable (annexin V) FC-DNT (CD3^+^TCRαβ^+^CD4^−^CD8^−^CD38^+^CD45RA^+^) were determined by flow cytometry after respective gating. Summary plot showing the survival of HD FC-DNT after addition of 2DG normalized to the Stim condition (cell count day 1*100/cell count day 1 Stim). In all figures, statistical comparisons were conducted using the one-way ANOVA test for multiple comparisons. Only statistically significant differences between subsets are shown (*P < 0.05, **P < 0.01, ***P < 0.001, and ****P < 0.0001). Bars and error bars indicate mean ± SD.

**Figure S4.**
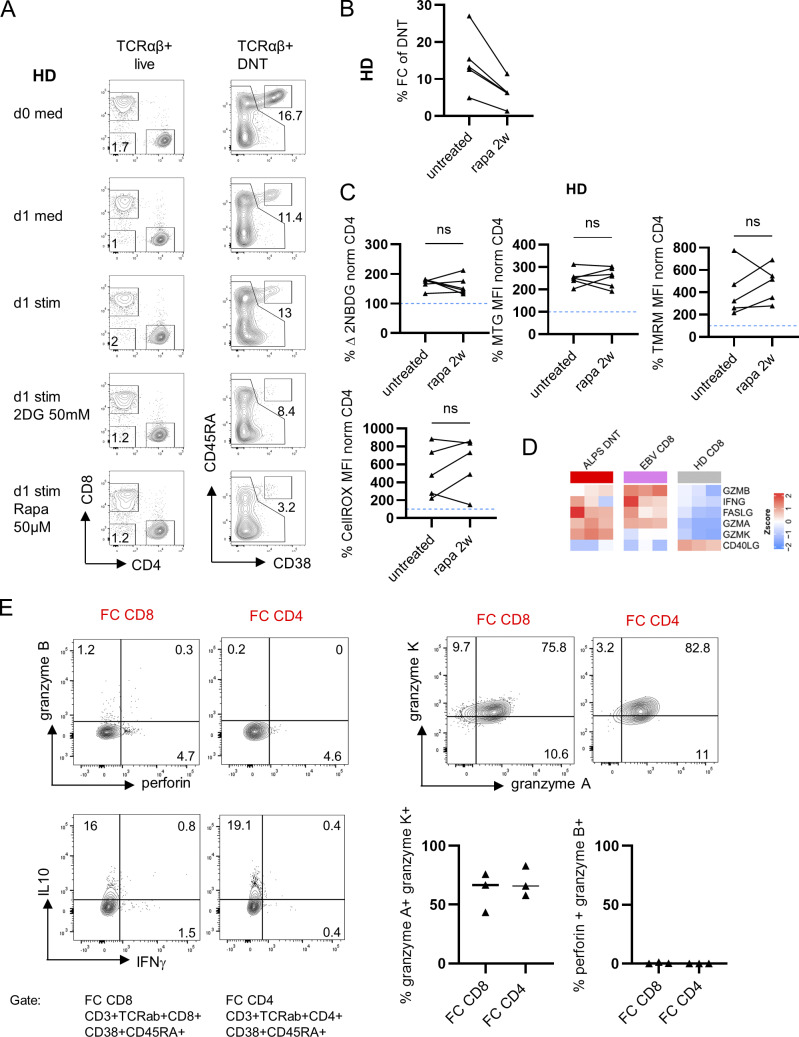
**Extended data on HD FCT survival, metabolic features and effector molecule production.** **(A)** Representative flow cytometry plots showing FC-DNT (CD38^+^CD45RA^+^) of an HD at day (d) 0 (immediately after blood withdrawal and PBMC isolation) and after 18 h of in vitro incubation with medium (Med), allogenic monocyte-derived DC at a ratio 1:1 plus IL-21 100 ng/ml and IL-10 100 ng/ml (Stim), or with the Stim condition plus 2DG or rapamycin (Rapa) at the indicated concentrations. **(B)** Summary graph showing FC-DNT (as % of total DNT) reduction in immunologically healthy individuals under treatment with rapamycin for 2 wk (2w). **(C)** Summary plot for 2-NBDG, MTG, TMRM, and CellROX median fluorescence intensities (MFIs) in FC-DNT of five immunologically healthy individuals under treatment with rapamycin normalized to the MFI of CD4 as in Fig. 2 A. Statistics were performed with one-way ANOVA test for multiple comparisons. **(D)** Transcriptional levels of selected effector molecules based on RNA-sequencing data from sorted ALPS-DNT (CD3^+^TCRαβ^+^CD4^−^CD8^−^), EBV-CD8 (CD3^+^CD8^+^CD38^+^), and HD-CD8 (CD3^+^CD8^+^) from three ALPS patients, three individuals with acute EBV infection, and three HD. The relative expression (Z-score) of genes is shown and color coded according to the legend. Rows are scaled to have a mean of 0 and SD of 1. **(E)** Representative flow cytometry plots showing granzyme B, A, and K, perforin, IL-10, and IFNγ expression in FC CD4 and FC CD8 of one ALPS patient and summary plots of three different ALPS patients.

We previously showed that FCT are prominent in ALPS patients but also exist in healthy individuals at low frequency with the same phenotype and the same clear-cut difference from other conventional T cell populations, including other DNT (1). The differentiation and transcriptional program of FCT are not restricted to DNT but can be found in small subsets of CD4 and CD8 T cells. For this reason, we also analyzed glucose uptake, glucose dependency, mitochondrial mass, MMP, and ROS production in FC-CD4, -CD8, and -DNT of healthy individuals. We used flow cytometry–based methods instead of Seahorse extracellular flux assays due to the small cell numbers. While in ALPS patients the majority of DNT carry the FCT signature, cells with this signature are rare in HD, below 1% among CD4 and CD8 and below 40% among DNT. We therefore specifically gated on CD38^high^CD45RA^high^ (FC) versus non-CD38^high^CD45RA^high^ “conventional” T cell populations for this analysis (Fig. 4 A). Interestingly, we found similarly elevated glucose uptake, increased mitochondrial mass, hyperpolarized mitochondria, and high ROS in all CD4, CD8, and DNT FCT subsets of HD as seen in their expanded FAS-deficient counterparts of ALPS patients (Fig. 4, B–E). FC-DNT of healthy individuals also showed impaired viability ex vivo and reduced survival upon glucose inhibition (Fig. 4 F and Fig. S4 A), but cell death as a result of 2-DG treatment was not as pronounced as in ALPS-DNT. In conclusion, the metabolic profile of FCT is intrinsic to this distinct T cell differentiation state and neither restricted to the DNT state nor to FAS deficiency.

### Rapamycin reduces aerobic glycolysis and survival of FCT but does not revert other hallmark metabolic features

**Figure 5.**
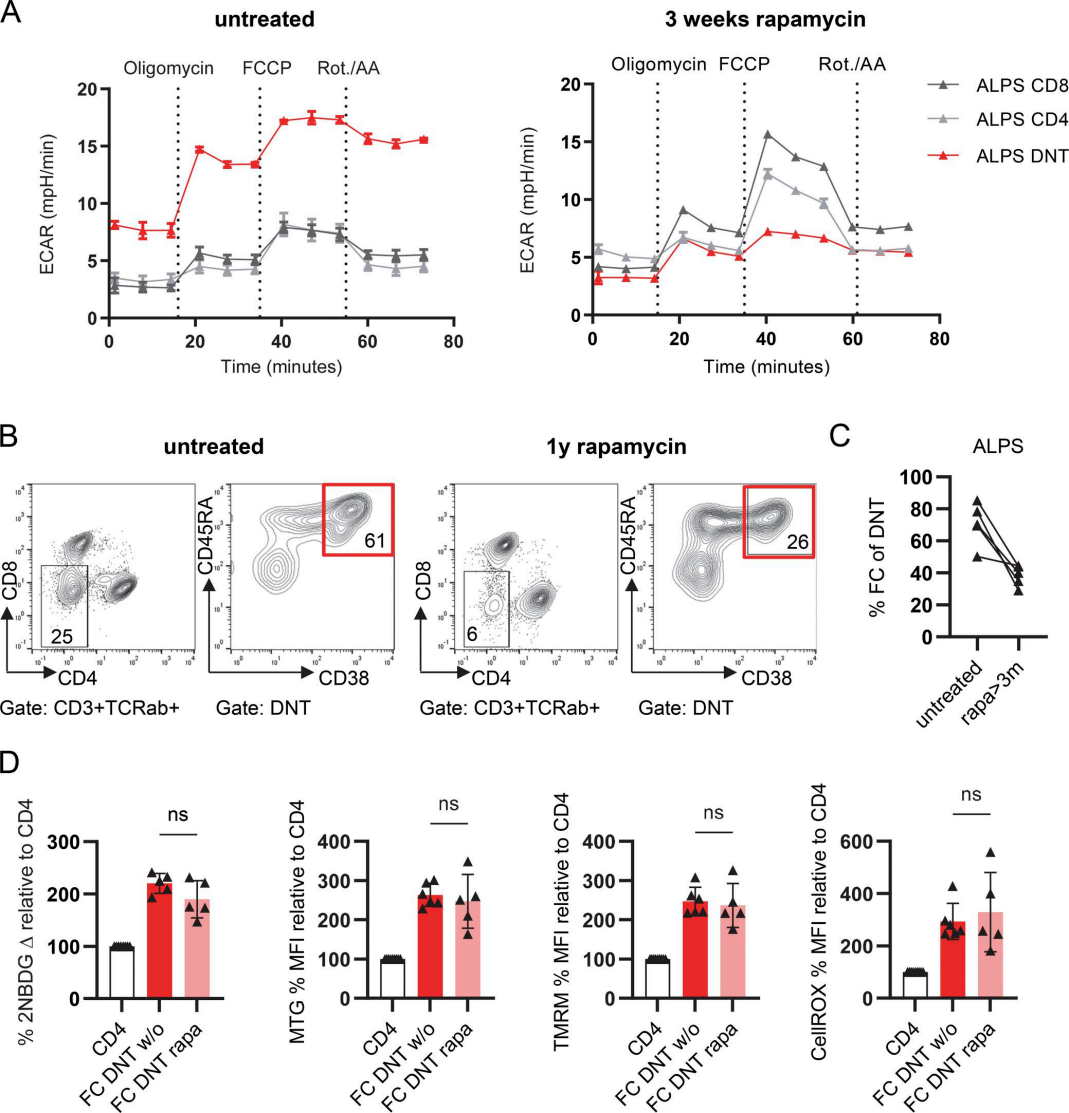
**Rapamycin reduces glycolysis and survival of FCT. (A)** Seahorse curves showing ECAR (see Fig. 2) of sorted ALPS-DNT (CD3^+^TCRαβ^+^CD4^−^CD8) or CD4/8 T cells before (left panel) and after 3 wk of rapamycin therapy (right panel). **(B)** Representative flow cytometry plots showing FC-DNT and other DNT among total TCRαβ^+^ DNT of one ALPS patient before and under rapamycin treatment (1 year). **(C)** Summary graph showing the percentages of CD38^+^CD45RA^+^ FC-DNT among total DNT before and after treatment with rapamycin for at least 3 mo in four ALPS patients. **(D)** Summary plots of delta 2-NBDG, MTG, TMRM, and CellROX median fluorescence intensities (MFIs) in FCT of five ALPS patients. The MFI values were normalized to that of CD4. Statistics with one-way ANOVA test for multiple comparisons.

DNT, and more precisely FC-DNT, rapidly decline in ALPS patients treated with the mTOR inhibitor rapamycin (15), which impacts many cellular processes, including glycolysis (18, 36). Since FCT survival and/or expansion is glucose metabolism dependent (Fig. 2, E and F), we hypothesized that the anti-glycolytic effect of rapamycin likely contributes to its efficacy. To address whether rapamycin reverts the metabolism of FCT to less glycolysis, we compared the glycolytic activity of ALPS-DNT sorted from one ALPS patient before and after 3 wk of clinically effective rapamycin treatment. DNT had declined from 9% to 6% of CD3 T cells. Interestingly, ECAR was no longer elevated in ALPS-DNT under rapamycin (Fig. 5 A). To address if rapamycin altered other metabolic features of FCT, we performed additional flow cytometry–based metabolic studies of DNT from ALPS patients before and under rapamycin. Rapamycin treatment reduced the frequency of FC-DNT among total ALPS-DNT (Fig. 5, B and C), but it did not change other metabolic features of the remaining FC-DNT, which continued to show elevated glucose uptake, mitochondrial mass, MMP, and ROS (Fig. 5 D). Similarly, in immunologically healthy individuals who received rapamycin for other diseases (Fig. S4, B and C), rapamycin reduced FC-DNT frequencies but did not change these investigated metabolic features. Thus, decreased glycolysis does not appear to be linked to reduced glucose/2NBDG uptake. In summary, mTOR inhibition impacts FCT survival and aerobic glycolysis but does not revert other hallmark metabolic features associated with FCT cell differentiation.

### Glycolytic metabolism is uncoupled from classical effector differentiation and function in FCT

**Figure 6.**
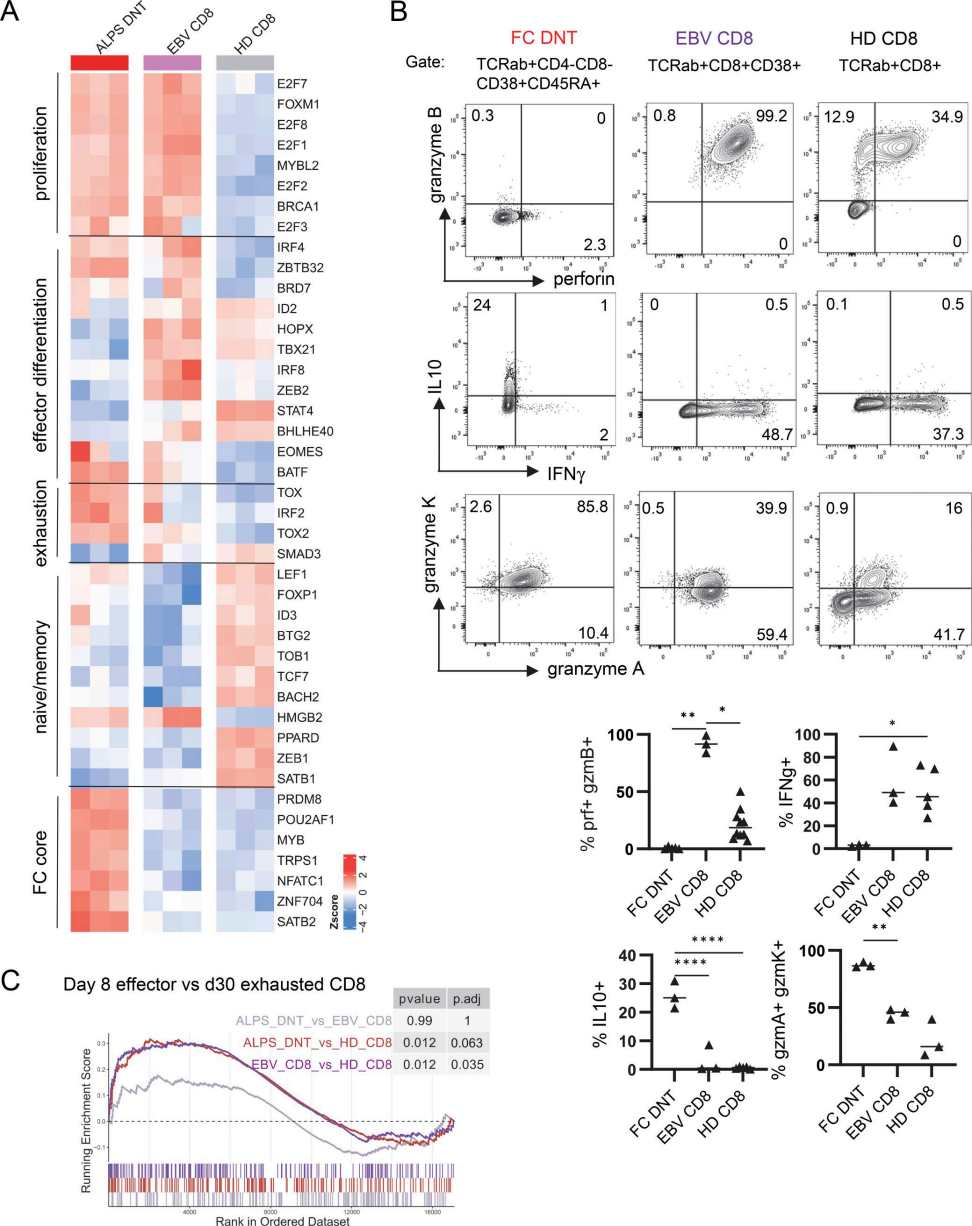
**Despite shared metabolic features with EBV-CD8, FCT do not show classical effector differentiation and function. (A)** Expression levels of selected TFs based on RNA-sequencing data from sorted ALPS-DNT (CD3^+^TCRαβ^+^CD4^−^CD8^−^), EBV-CD8 (CD3^+^CD8^+^CD38^+^), and HD-CD8 (CD3^+^CD8^+^) from three ALPS patients, three individuals with acute EBV infection, and three HD. The relative expression (Z-score) of genes is shown and color coded according to the legend. Rows are scaled to have a mean of 0 and SD of 1. **(B)** Representative flow cytometry plots and summary plots showing production of the indicated cytokines or effector molecules in FC-DNT of ALPS patients (*n* = 3), EBV-CD8 (*n* = 3), and HD-CD8 (*n* = 3–9) after stimulation of total PBMC with PMA/ionomycin for 5 h and subsequent gating. Statistics with one-way ANOVA test for multiple comparisons (*P < 0.05, **P < 0.01, and ****P < 0.0001). **(C)** Enrichment score plot generated with RNA-sequencing data of the indicated sorted T cell types using the gene set of day 8 acute LCMV CD8 effector versus day 30 chronic (clone 13) LCMV-exhausted CD8 T cells published in (41) (GSE41867) (NES: ALPS-DNT vs. EBV-CD8: 0.70; ALPS-DNT vs. HD-CD8: 1.36; EBV-CD8 vs. HD-CD8: 1.33).

TCR activation in combination with CD28 co-stimulation enhances mTOR signaling and induces metabolic remodeling, including an increase in glycolysis, which has been directly linked to effector differentiation and function. While cell cycle–related TFs were similarly expressed by ALPS-DNT and EBV-CD8, only the latter showed high transcriptional levels of the TFs TBX21, ZEB2, and IRF8, which are typically expressed by CD8 effector T cells (Fig. 6 A). However, the expression of other effector and exhaustion-associated TFs, including EOMES, BATF, TOX/TOX2, and IRF2, was even higher in FCT than in EBV-CD8 (37, 38, 39, 40). Notably, FCT highly expressed TFs, including MYB, POU2AF1, PRDM8, and NFATC1, which in this combination clearly delineates them from other T cell populations. Strikingly, while IFNG transcript levels were elevated in FCT (Fig. S4 D), they were not able to produce IFNγ after stimulation with PMA/ionomycin (Fig. 6 B) ex vivo, and expression of perforin and granzyme B was almost absent (Fig. 6 B) (shown also in 1). In contrast, they produced high amounts of IL-10 and granzyme K (Fig. 6 B). This functional signature was shared by FC-CD4 and FC-CD8 (Fig. S4 E). Interestingly, exhausted CD8 T cells, best described in murine models of chronic LCMV infection, are also characterized by a dysfunctional effector state. To further position FCT between effector and exhausted CD8 T cells, we performed a gene set enrichment analysis using our human RNA-sequencing data and murine reference data of day 8 acute LCMV CD8 effector versus day 30 chronic (clone 13) LCMV-exhausted CD8 T cells (41). Both EBV-CD8 and FCT showed an enrichment of genes associated with acute effector rather than exhausted CD8 T cells (Fig. 6 C). Hence, FCT differentiation is associated with an effector-like glycolytic switch and high proliferative activity but remains uncoupled from classical effector differentiation. The FCT state is characterized by a distinct TF profile along with a more undifferentiated state regarding classical effector functions.

## Discussion

In this study, we dissect the metabolic programs that drive pathological T cell proliferation in ALPS—a disease in which highly proliferative, FCT evade apoptosis. We compared these FCT to CD8 T cells in acute EBV infection, a model of physiological, though sometimes excessive, antigen-driven T cell expansion. While both conditions are marked by T cell–mediated lymphadenopathy and splenomegaly, the identity of the disease-driving T cells and the nature of their activating triggers differ. By comparing these two conditions, we identified overlapping and distinct metabolic features with potential relevance for therapeutic intervention. By assessing genome-scale metabolic models reflective of the distinct metabolic wiring of the investigated T cell populations, we offer a resource to complement experimental and clinical research with in silico analyses capable of estimating metabolic pathway activities or flux states under various perturbations and conditions. These in silico analyses are useful to either support existing experimental findings or generate new hypotheses that need to be validated through further experiments.

Unexpectedly, we found that FCT engage a metabolic program as strongly glycolytic as that of acutely activated, virus-specific CD8 T cells during primary EBV infection. This is remarkable, given that EBV-driven T cells are often considered the benchmark for rapid proliferation and effector function—optimally tuned to mount a vigorous antiviral response. Yet, despite lacking a known antigenic stimulus, FCT showed equally high proliferative activity, elevated activation markers, active mTOR pathway signaling, and markedly increased glucose metabolism. Clinical observations have hinted at this glycolytic bias, as ALPS patients often show enhanced 18F-fluorodeoxyglucose Positron emission tomography (PET) positivity in enlarged lymph nodes (42, 43). Similarly, in Fas-deficient MRL/lpr mice, DNT exhibit higher baseline glycolysis than CD4 or CD8 T cells (44, 45). However, without comparison to a well-defined in vivo–activated T cell subset, the magnitude of glycolytic activity in FCT remained unclear. Here, we demonstrate that human FCT are as highly glycolytic as EBV-stimulated CD8 T cells. These findings suggest that uncontrolled T cell expansion relies on a shared, augmented glycolytic state. In vitro, FCT displayed a strong decrease in survival upon glucose metabolism inhibition, which was mirrored in the in silico analysis. This effect may not only reflect glucose dependency but possibly also decreased capacity of ALPS-DNT to utilize alternative metabolic pathways, an intrinsically higher susceptibility of ALPS-DNT to cell death and/or reduced functionality of glucose deprived moDCs in our experimental setting.

One of our main observations is that, despite their bioenergetic similarity to classical effector T cells, FCT fail to execute canonical effector differentiation programs. Whereas EBV-responsive CD8 acquire potent cytotoxic function, FCT, including FC-CD8 and their FC-DNT progeny, do not produce IFNγ, granzyme B, or perforin and thus remain functionally uncoupled from the classical CD8 effector cytokine and cytolytic mediator production. In murine effector T cells, glycolytic engagement of Ldha and Gapdh allows IFNγ transcription and translation, respectively (46, 47), suggesting a direct link between metabolism and effector function. How this mechanism relates to the proliferation–effector uncoupling in FCT remains to be clarified. Our results indicate that the usual association of mTOR activation, glycolysis, and effector differentiation does not apply across all proliferative CD8-derived subsets. In FCT, mTOR and glycolysis support a distinct differentiation program with an altered functional profile. Moreover, the combination of increased MYB and absent T-BET expression could play a role in preventing classical effector differentiation in FCT (48). Notably, increased glycolysis upon activation can also be linked to IL-4 or IL-17 instead of IFNγ production in the respective CD4 T helper subsets. Thus, not only metabolic cues but also T cell–intrinsic and –extrinsic factors, including mode of antigen encounter and cytokines, determine differentiation and effector profile. Interestingly, regulatory T cells that also highly express IL-10 exhibit only a partial metabolic shift to glycolysis upon activation—though not as robust as conventional effector T cells. FAO remains their dominant metabolic signature and is essential for lineage stability and their core regulatory functions (49, 50).

We also identify metabolic features unique to FCT, including markedly elevated MMP and ROS, absent in EBV-CD8. Consistent with reports of enlarged, rounded mitochondria in DNTs from MRL/lpr mice (45), human FCT displayed increased mitochondrial fission and altered cristae structure, with potential consequences for mitochondrial function (25). Nevertheless, OXPHOS was largely intact as indicated by comparable basal OCR. Previous studies in MRL/lpr mice even showed enhanced OXPHOS in DNT compared to CD4 and CD8 T cells (45). In our study, human FCT dying in vitro showed a rapid decrease of both the MMP and ROS levels, indicating that mitochondria represent a major source of ROS in FCT. When the MMP is high, electrons tend to back up in complexes I and III, increasing the probability of electron leakage to molecular oxygen, generating ROS. In addition, insufficient antioxidant capacity as suggested by our in silico analyses may contribute to the elevated cellular ROS but requires experimental validation (51, 52). ROS are known to promote activation and expansion of antigen-specific T cells (28) and act as signaling intermediates (53). Among ROS-responsive elements, HMOX1 and NFATC1 were particularly upregulated in FCT. However, excessive ROS can be cytotoxic, possibly contributing to the rapid ex vivo death of FCT and potentially to their short lifespan in vivo. In line with this, high MMP was shown to correlate with short-lived, non–self-renewing phenotypes, while low MMP is associated with stemness and longevity (35). Assuming a detrimental role of ROS, it would be interesting to explore the effects of ROS scavenging. However, mitochondrial ROS scavenging did not alter DNT frequency or lymphoproliferation in a mouse model of Fas deficiency (45), suggesting either the inefficiency of the scavenger used or the insufficiency of ROS scavenging in restoring viability in FCT. Chronic antigen-experienced exhausted CD8 T cells also exhibit elevated ROS and NFATC1, and FCT share some features with these cells, including upregulation of TOX, PD-1, CTLA-4, and LAG-3 (1, 54). However, FCT differ strikingly by their complete lack of T-BET, high expression of MYB and POU2AF1, sustained proliferation, and high IL-10 production—features not typical of exhausted cells. Moreover, FCT are highly glycolytic with increased MMP, whereas exhausted T cells generally show reduced glycolysis and depolarized mitochondria (24).

The highly glycolytic signature in combination with hyperpolarized mitochondria defines a distinct T cell differentiation state found in subsets of CD4, CD8, and DNT in both ALPS patients and healthy individuals. This suggests that FCT represent a physiologically relevant subset characterized by an intrinsic mTOR-driven glucose-dependent anabolic program. The signals that initiate this state and drive lymphoproliferation remain unknown but may include self-antigens. Our data suggest that these signals induce anabolic metabolism and proliferation but are not sufficient or processed in a different signaling context that fails to promote classical effector differentiation. Instead, they initiate a separate differentiation trajectory supported by a unique TF profile. In healthy individuals, FCT are tightly regulated and rapidly eliminated via FAS-mediated apoptosis. Hence, their life cycle appears to be designed for maximal, transient proliferative bursts at the expense of stemness and longevity. In ALPS, loss of the apoptotic FAS checkpoint permits uncontrolled expansion of these metabolically primed but functionally altered T cells, driving pathological lymphoproliferation without significant inflammation.

The glucose dependency of FCT survival could offer a rationale for targeted metabolic therapy. We show that glycolysis blockade with 2DG rapamycin strongly impairs FCT survival. This observation is clinically relevant, as rapamycin is already used therapeutically for ALPS, consistent with the observation that FCT are “addicted” to anabolic, mTOR-driven metabolism. Interestingly, unlike certain exhausted CD8 subsets (24), FCT partially retain an altered metabolic profile under rapamycin treatment, suggesting that rapamycin primarily limits survival rather than fully reprogramming FCT metabolism. Future studies should aim to define the stimuli that initiate and sustain FCT proliferation and explore additional metabolic interventions for targeted immunomodulation in ALPS and related lymphoproliferative disorders.

## Materials and methods

### Patients and healthy individuals

We included 24 ALPS-FAS/sFAS patients and 13 patients without known genetic predisposition, who presented with lymphadenopathy and splenomegaly in the context of serologically confirmed acute primary EBV infection. Table S1 summarizes genetic and clinical characteristics, including age and gender. Moreover, five immunologically healthy individuals before and after rapamycin treatment (mainly for vascular malformations) were analyzed. Finally, age-matched healthy individuals were analyzed in parallel. Experiments were conducted primarily using fresh blood samples of untreated individuals, which were processed on the day of blood sampling to accurately capture the in vivo cellular state. All participants gave informed consent. The study was conducted according to the Declaration of Helsinki and approved by Ethics Committees of the University of Freiburg (protocol numbers: 409/16, 282/11, 610/15, and 147/15).

### Flow cytometry and cell sorting

Flow cytometry was performed with PBMC. The following antibodies were used: CD3 PerCP (clone SK7; BD Biosciences; RRID: AB_400513), CD8 BV510 (clone RPA-T8; BD Horizon; RRID: AB_314120), CD8 BUV395 (clone RPA-T8; BioLegend; RRID: AB_893425), CD8 PC7 (clone SFCI21Thy2D3; Beckman Coulter), CD8 Krome Orange (PN B00067; Beckman Coulter), TCRαβ FITC (clone T10B9.1A-31; BD Biosciences), TCRαβ BV421 (clone IP26; BioLegend), TCRαβ PE (clone IP26A; Beckman Coulter), CD45RA PC7 (clone HI100; BD Biosciences), CD45RA BV510 (clone HI100; BioLegend), HLA-DR PC7 (clone L243/G46-6; BD Pharmingen), HLA-DR FITC (clone G46-6; BD Biosciences), Ki-67 PC7 (clone B56; BD Biosciences), Ki-67 FITC (clone MIB-1; DAKO), CD4 APC-Cy7 (clone RPA-T4; BioLegend), CD4 PB (clone 13B8.2; Beckman Coulter), CD4 PE (clone 13B8.2; Beckman Coulter), CD4 FITC (clone RPA-T4; BD Pharmingen), CD4 APC (clone SK3; BD Pharmingen), CD38 PerCP (clone HIT2; BioLegend), CD38 APC (clone HIT2; BioLegend), CD38 BV421 (clone HB-7; BioLegend), T-BET PE (clone eBio4B10; eBioscience), EOMES APC (clone WD1928; eBioscience), IFN-γ FITC (clone B27; BD Biosciences), IL-10 PE (clone JES3-9D7; BioLegend), Perforin FITC (clone delta G9; Ancell), Granzyme B BV421 (clone GB11; BD Biosciences), Granzyme A PC7 (clone CB9; BioLegend), Granzyme K FITC (clone GM26E7; BioLegend), GLUT1 AlexaFluor647 (clone EPR3915; Abcam), HIF1α PE (clone 546-16; BioLegend), Annexin V FITC (BD Pharmingen), Annexin V BV605 (BD Horizon), and Annexin V APC (BD Pharmingen). Intracellular GLUT1 and HIF1α stainings were performed using the BD Cytofix/Cytoperm Kit. GLUT1 surface staining was performed with GLUT1-G125 (GLUT1 cell surface RBD ligand Kit, METAFORA biosystems). Perforin, granzyme B, Ki-67, and Eomes stainings were performed using the Human Regulatory T cell Staining Kit (eBioscience). PBMC were incubated with 2-NBDG-FITC (Life Technologies), MitoTracker Green (Thermo Fisher Scientific), TMRM (Thermo Fisher Scientific), and CellRox (Invitrogen) for 15 min at 37°C. To measure cytokine production, 4 × 10^6^ PBMC were stimulated with PMA (Sigma-Aldrich, 3.5 µg/ml) and ionomycin (Sigma-Aldrich, 2 µM) for 5 h and then analyzed using the Cytofix/Cytoperm Plus Kit and Golgi Plug (BD). Data were analyzed using FlowJo 7.2.5 or 10.4 software (Tree Star). Cell populations were either magnetically enriched using the Double-negative T cell Isolation Kit or the CD8 Isolation Kit (both Miltenyi Biotec) or sorted with MoFlo Astrios (Beckman Coulter) or FACS Aria Fusion (Becton Dickinson) cell sorters immediately after PBMC isolation on the day of blood collection.

### Extracellular flux analysis: Seahorse assay

ECAR and OCR were measured using the Seahorse XFe 96 bioanalyzer (Agilent) as previously described (55). 2 × 10^5^ MACS magnetic-activated cell sorting (MACS) enriched or sorted T cells per well (3 wells per sample) were spun onto poly-D-lysine–coated seahorse 96-well plates and preincubated at 37°C for a minimum of 45 min in the absence of CO_2_. OCR and ECAR were measured under basal conditions and after the addition of the following drugs: 1 mM oligomycin, 1.5 mM flurorcarbonyl cynade phenylhydrazon, and 100 nM rotenone + 1 mM antimycin A (all Sigma).

### Survival and proliferation assay

For the survival assay 2 × 10^5^ PBMC per well were seeded into 96-well plates and stimulated either with medium alone or with allogenic monocyte-derived DC (moDC) (ratio 1:1, generated using the moDC Generation toolbox from Miltenyi Biotec) plus IL-21 (100 ng/ml, Miltenyi Biotec) and IL-10 (100 ng/ml, PreproTech) for 18 h at 37°C. As metabolic intervention, 2-DG (Sigma-Aldrich) or rapamycin (Selleckchem) was added at different concentrations. Survival was measured by flow cytometry, and annexin V staining was performed using the Annexin V binding buffer (BD Pharmingen). Survival normalized to day 0 was calculated as (cell count day 1/cell count day 0)*100. Survival normalized to the Stim condition was calculated as (cell count day 1*100/cell count day 1 Stim). For the proliferation assay, PBMCs were labeled with CFSE (Molecular Probes, C-1157), seeded into 96-well plates, and stimulated either with medium alone or with plate-bound CD3 OKT3 (14-0037; eBioscience) and soluble CD28 (555725; BD) in the presence of increasing concentrations of rapamycin (Selleckchem). Proliferation was measured after 5 days via flow cytometry.

### RNA-sequencing analysis

DNT (CD3^+^TCRαβ^+^CD4^−^CD8^−^CD38^+^CD45RA^+^) of three ALPS-FAS patients, CD8 T cells (CD3^+^CD8^+^CD28^+^CD57^−^) of three HD (peripheral blood buffy coats), and CD8 T cell (CD3^+^CD8^+^CD38^+^) of three individuals with acute EBV infection (two peripheral blood samples and one bone marrow blood sample) were sorted. RNA-sequencing steps included RNA isolation (Qiagen RNeasy microkit), RNA-quality analysis (2100 Bioanalyzer, Agilent Technologies), creation of barcoded RNA-sequencing libraries (from 100 ng total RNA with Ovation Human RNA-Seq system, Nugen), and depletion of rRNAs (insert-dependent adaptor cleavage method, Nugen). Alignment and quantification were performed as in (1). Downstream analyses were performed using R 4.4.0 (R Core Team 2015). Low expressed genes (total raw counts across the 15 samples ≤5) were filtered out, and differential expression analysis was performed with the DESeq package v.1.44 (RRID:SCR_000154). Significantly regulated genes were selected based on their adjusted P value (Benjamini and Hochberg) <0.05. All genes presented in the figures of this manuscript meet this significance threshold. Gene set enrichment was performed using clusterProfiler package v.4.12.6 (RRID:SCR_016884) with MSigDB gene sets v7.0. Adjusted P value <0.05 was considered as significant. Data are available under GEO Accession viewer (GSE154929 and GSE319376).

### In silico analysis of metabolic activity

A graphical summary of the workflow used for the analysis is provided in Fig. S1. We processed transcriptomic data from three T cell types (ALPS-DNT, EBV-CD8, and resting HD-CD8), each with three biological replicates, by generating mean expression profiles and mapping them to the Recon3D metabolic model (29). Using these profiles and a core set of 25 reactions, curated from a whole-body model (56) to include reactions expected to be active in CD8 T cells, as input, we reconstructed context-specific models via the fastCore algorithm (30) within the COBRA Toolbox v3.0 (57). These models were subsequently constrained with a simulated physiological T cell medium and further refined using the eFlux algorithm (30) based on gene expression data. Note that the only difference between the three cell type models is constituted by the input transcriptome data. All other aspects of the modeling, including the simulated medium, in silico experimental procedures, and settings within the fastCore algorithm, are identical across the three conditions.

A series of metabolic flux analyses were then performed, including FBA to predict maximum biomass under unconstrained, physiologically constrained, and eFlux-constrained conditions and flux variability analysis (FVA) to delineate the feasible solution space at optimal biomass. We also assessed the relative contributions of glycolysis and OXPHOS to ATP production, simulating anoxia by blocking oxygen uptake and impairing glycolysis by blocking hexokinase reaction. We further simulated FAO inhibition by blocking all reactions facilitated by CPT1, PPP (Pentose Phosphate Pathway) inhibition by blocking the enzyme G6PD, and blocking glutaminolysis by either blocking glutamine uptake or the mitochondrial glutaminolysis. Finally, the optGpSampler (58) algorithm generated 10,000 flux samples per model to characterize probable flux distributions, and we repeated the random sampling using a second sampling algorithm (Riemannian Hamiltonian Monte Carlo [RHMC] sampling [https://doi.org/10.48550/arXiv.2202.01908]) for sensitivity analysis. Results in key reactions were comparable across the two sampling algorithms. Key reactions were visualized using kernel density estimation and box plots. To test in silico the oxidative stress response, we induced 2% superoxide production per consumed mmol oxygen in the mitochondrial electron chain in all three models. Then, we investigated superoxide leakage and superoxide detoxification pathways via random sampling with the RHMC sampling, setting 10,000 samples as the target. Finally, we assessed NADPH production capacity by adding a NADPH sink reaction to the models and performing FVA. Context-specific metabolic models, full FVA results, random sampling, all scripts, and the complete modeling output can be accessed via https://github.com/SysPsyHertel/CodeBase/tree/main/Scripts_Huang_Tcell.

### Electron microscopy

Sorted T cells (1.2  ×  106 DNT of an ALPS patient and 2  ×  10^6^ CD3 of two healthy individuals) were fixed in 2.5% glutaraldehyde in 100 mM sodium cacodylate and washed in cacodylate buffer. After dehydration, samples were embedded in Eponate 12 resin (Ted Pella), and sections were cut. Images were acquired using a FEI Tecnai 12 Transmission electron microscope equipped with a Veleta digital camera. Brightness and contrast were adjusted using ImageJ 1.44o (NIH) (RRID:SCR_003070). The analysis was performed blindly and using ImageJ 1.44o (NIH): only entire mitochondria were included in the analysis; ratio length/width, maximal cristae width, and numbers of cristae per mitochondrion were measured.

### Statistical analysis

Statistical analyses were performed with GraphPad Prism software (RRID:SCR_002798). *T* tests (Mann–Whitney U), Wilcoxon test or ANOVA, Kruskal–Wallis test for multiple comparisons, and mixed-effects analysis with Tukey's multiple comparisons test were applied to compare T cell marker expression or frequencies (*P < 0.05, **P < 0.01, ***P < 0.001, and ****P < 0.0001). If a comparison was not significant, no sign or ns was added. To allow for comparisons across multiple acquisition dates of the flow cytometry data, we normalized the data to the values of the CD4 subset of the same individual.

### Online supplemental material

The supplemental material includes one table and four figures. Fig. S1 shows GLUT1 surface expression and the workflow for the generation and analysis of context-specific metabolic models in silico. Figs. S2 and S3 extend data on glycolytic and OXPHOS metabolism and on survival assays. Fig. S4 adds metabolic data on FCT in HD and on the analysis of the effector profile of FCT. Table S1 shows the clinical and genetic features of the individuals involved in the study.

## Supplementary Material

Table S1 shows individuals involved in the study.

## Data Availability

The data that support the findings of this study are available from the corresponding author upon reasonable request. The RNA-sequencing data are available under GEO Accession viewer (GSE154929 and GSE319376). All in silico metabolic models generated in this study and the corresponding analysis scripts can be downloaded freely from https://github.com/SysPsyHertel/CodeBase/tree/main/Scripts_Huang_Tcell.
