# Supplementary material for: FAS-controlled T cells drive lymphoproliferation through glycolysis without effector differentiation
Source: J Hum Immun. 2026 May 13;2(4):e20250233. doi: 10.70962/jhi.20250233 (PMC13170455; doi:10.70962/jhi.20250233)
Supplement: Table S1 — shows individuals involved in the study. [file jhi_20250233_tables1.docx]

**Table S1. Individuals involved in the study**

| **Pt** | **Diagnosis** | **Age (y)** | **Gender** | **Symptoms** | **Mutation** | **%DNT**  **/CD3** | **sFasL pg/ml** | **%HLA-DR**  **/CD8** |
| --- | --- | --- | --- | --- | --- | --- | --- | --- |
| 1 | ALPS-FAS | 38 | F | Lp | *FAS* Exon 9 c.817C>T het | 10.9 | 1224 |  |
| 2 | ALPS-FAS | 5 | M | Lp | *FAS* Exon 9 c.817C>T het | 37 | 5131 |  |
| 3 | ALPS-FAS | 18 | M | Lp | *FAS* Exon 2 c.46-47delGC het | 8.6 | 1134 |  |
| 4 | ALPS-U | 9 | F | Lp, cytopenia | Hidden FAS | 15.6 | 4660 |  |
| 5 | ALPS-FAS | 33 | M | Lp | *FAS* Exon 2 c.46_47delGC het | 7.8 | 746 |  |
| 6 | ALPS-FAS | 8 | M | Lp | *FAS* Exon 3 c.277_280delGACA het | 14.3 | 5988 |  |
| 7 | ALPS-FAS | 13 | M | Lp | *FAS* Exon 9 c.749G>A het | 19 | 1553 |  |
| 8 | ALPS-FAS | 3 | F | Lp, cytopenia | *FAS* Duplication | 13.3 | 13616 |  |
| 9 | ALPS-FAS | 23 | M | Lp | Het. deletion from intron 2 of FAS | 8.7 | 6654 |  |
| 10 | ALPS-FAS | 17 | M | Lp | *FAS* IVS4 c.443+2T>C donor splice site het | 8.1 | 2952 |  |
| 11 | ALPS-FAS | 6 | M | Lp | *FAS* IVS2 c.196+1G>A donor splice site het | 7.8 | 653 |  |
| 12 | ALPS-sFAS | 11 | M | Lp, cytopenia | Somatic *FAS* IVS7 c.652-1G>A het | 16 | 1560 |  |
| 13 | ALPS-FAS | 28 | M | Lp | *FAS* Exon 6 c.536T>G het | 12.6 | 1796 |  |
| 14 | ALPS-FAS | 18 | F | Lp, cytopenia | *FAS* Exon 3 c.(254G>T( - )255C>T) het | 5.4 | 865 |  |
| 15 | ALPS-FAS | 17 | F | Lp | *FAS* Exon 9 c.779A>G het | 18 | 1966 |  |
| 16 | ALPS-FAS | 15 | M | Lp, cytopenia | FAS Exon 9 c.703delA het | 5 | 4084 |  |
| 17 | ALPS-FAS | 6 | M | Lp, cytopenia | FAS IVS7 c.651+1G>A donor splice site het | 18 | 3532 |  |
| 18 | ALPS-FAS | 6 | M | Lp, cytopenia | *FAS* Exon 2 c46_47delGC het | 13 | 3650 |  |
| 19 | ALPS-FAS | 3 | M | Lp, cytopenia | *FAS* Exon 2 c46_47delGC het | 29 | 8643 |  |
| 20 | ALPS-sFAS | 26 | F | Lp | Somatic *FAS* Exon 8 c.999-1001delA het | 7 | 639 |  |
| 21 | ALPS-FAS | 16 | F | Lp | *FAS* Exon 6 c.568G>T het | 9.5 | 1494 |  |
| 22 | ALPS-FAS | 4 | F | Lp | *FAS* Exon 9 c.748C>T het | 5.7 | 5241 |  |
| 23 | ALPS-FAS | 24 | M | Lp | *FAS* Exon 4 c.404del het | 8.6 | 4863 |  |
| 24 | ALPS-FAS | 24 | M | Lp | *FAS* Exon 9 c.779A>G het | 27.2 | 1964 |  |
| 1 | EBV | 8 | F | Secondary HLH |  | 0.6 | 819 | 91 |
| 2 | EBV | 6 | M | Tonsillitis, cytopenia |  | 1.2 | 1344 | 81 |
| 3 | EBV | 17 | F | Lp, fever, tcpenia, tonsillitis |  | 0.7 | n.a. | 77 |
| 4 | EBV | 17 | M | Fever, tonsillitis, lp |  | 0.9 | n.a. | 85 |
| 5 | EBV | 16 | F | Fever, tcpenia, tonsillitis |  | 0.7 | 685 | 87 |
| 6 | EBV | 7 | M | Fever, tonsillitis, lp |  | 0.6 | 1449 | 91 |
| 7 | EBV | 17 | F | Fever, tcpenia, lp |  | 1.4 | 655 | 86 |
| 8 | EBV | 18 | F | Tonsillitis |  | 1 | n.a. | 79 |
| 9 | EBV | 15 | M | Tonsillitis, lp |  | 0.7 | n.a. | 85 |
| 10 | EBV | 15 | F | Fever, lp, tcpenia |  | 0.9 | n.a. | 93 |
| 11 | EBV | 2 | F | Fever, tonsillitis, lp |  | 1.2 | 1448 | 75 |
| 12 | EBV | 51 | M | Secondary HLH |  | 1.1 | n.a. | 60 |
| 13 | EBV | 17 | F | Secondary HLH |  | 0.1 | n.a. | 76 |

Lp: lymphoproliferation (splenomegaly and/or lymphadenopathy). Tcpenia: thrombocytopenia. HLH: haemophagocytic lymphohistiocytosis. n.a.: not available. Normal range sFasL < 200 pg/ml. Normal range CD4-CD8-TCRαβ+/CD3 < 2.5%. All EBV patients had IgM positive for EBV or positive EBV PCR.
